# Disease-related cellular protein networks differentially affected under different *EGFR* mutations in lung adenocarcinoma

**DOI:** 10.1038/s41598-020-67894-7

**Published:** 2020-07-02

**Authors:** Toshihide Nishimura, Haruhiko Nakamura, Ayako Yachie, Takeshi Hase, Kiyonaga Fujii, Hirotaka Koizumi, Saeko Naruki, Masayuki Takagi, Yukiko Matsuoka, Naoki Furuya, Harubumi Kato, Hisashi Saji

**Affiliations:** 1 Department of Translational Medicine Informatics, St. Marianna University School of Medicine, Kawasaki, Kanagawa 216-8511 Japan; 2 Department of Chest Surgery, St. Marianna University School of Medicine, Kawasaki, Kanagawa 216-8511 Japan; 3 The Systems Biology Institute, Tokyo, 141-0022 Japan; 4 Department of Pathology, St. Marianna University Hospital, Kawasaki, Kanagawa 216-8511 Japan; 5 Division of Respiratory Medicine, Department of Internal Medicine, St. Marianna University School of Medicine, Kawasaki, Kanagawa 216-8511 Japan; 6 Tokyo Medical University, Tokyo, 160-0023 Japan; 7 International University of Health and Welfare, Tokyo, 107-8402 Japan

**Keywords:** Cancer, Cell biology, Computational biology and bioinformatics, Drug discovery, Immunology, Systems biology, Biomarkers, Medical research, Molecular medicine, Oncology

## Abstract

It is unclear how epidermal growth factor receptor *EGFR* major driver mutations (L858R or Ex19del) affect downstream molecular networks and pathways. This study aimed to provide information on the influences of these mutations. The study assessed 36 protein expression profiles of lung adenocarcinoma (Ex19del, nine; L858R, nine; no Ex19del/L858R, 18). Weighted gene co-expression network analysis together with analysis of variance-based screening identified 13 co-expressed modules and their eigen proteins. Pathway enrichment analysis for the Ex19del mutation demonstrated involvement of SUMOylation, epithelial and mesenchymal transition, ERK/mitogen-activated protein kinase signalling via phosphorylation and Hippo signalling. Additionally, analysis for the L858R mutation identified various pathways related to cancer cell survival and death. With regard to the Ex19del mutation, ROCK, RPS6KA1, ARF1, IL2RA and several ErbB pathways were upregulated, whereas AURK and GSKIP were downregulated. With regard to the L858R mutation, RB1, TSC22D3 and DOCK1 were downregulated, whereas various networks, including VEGFA, were moderately upregulated. In all mutation types, CD80/CD86 (B7), MHC, CIITA and IFGN were activated, whereas CD37 and SAFB were inhibited. Costimulatory immune-checkpoint pathways by B7/CD28 were mainly activated, whereas those by PD-1/PD-L1 were inhibited. Our findings may help identify potential therapeutic targets and develop therapeutic strategies to improve patient outcomes.

## Introduction

Mutations in the tyrosine kinase domain of the epidermal growth factor receptor (*EGFR*) were identified as causes of non-small-cell lung cancer (NSCLC) in 2004^1,2^. Somatic mutations in the kinase domain of the *EGFR* gene are detected in approximately 40% and 17% of lung adenocarcinomas in Asians^3^ and Caucasians^4^, respectively. The most common oncogenic mutations are small, in-frame deletions in exon 19 (44.8%) and a point mutation that substitutes Leu-858 with arginine (L858R) (39.8%)^5^. Importantly, activating mutations have been found to confer sensitivity to the small molecule tyrosine kinase inhibitors (TKIs) gefitinib, erlotinib and afatinib. These EGFR-TKIs (targeted therapies for patients with *EGFR*-mutant NSCLC) have been established as standard first-line treatments according to pivotal phase III trials that reported an improved objective response rate of approximately 70% and significantly longer progression-free survival (PFS) (range, 8.0–13.7 months) with EGFR-TKIs than with conventional chemotherapy^6–8^. Eventually, drug resistance occurs in most patients after 1 year of treatment. Therefore, novel treatment strategies have been challenged to improve the survival benefit of first-line treatment. Basically, the efficacy of these EGFR-TKIs is limited based on the result of drug resistance conferred by another mutation involving substitution of threonine 790 with methionine (T790M)^9^. Osimertinib is an irreversible third-generation EGFR-TKI that is selective for sensitising EGFR and T790M mutations. The randomised phase III AURA3 trial demonstrated that the efficacy of osimertinib was significantly greater than that of platinum therapy plus pemetrexed in patients with T790M-positive advanced NSCLC^10^. The need for tissue re-biopsy to determine the T790M status can be a barrier to appropriate treatment selection. Plasma detection and semi-quantitation of the activating EGFR and T790M mutations are useful to predict the efficacy of osimertinib^11^. T790M in circulating tumour DNA was approved by the US FDA in 2016 as a diagnostic tool to predict osimertinib success, and minimally invasive assays are expected to gain prominence in the future. Recently, osimertinib was recommended as first-line treatment for patients with *EGFR*-mutant NSCLC according to the FLAURA trial that reported significantly better PFS and OS with osimertinib than with first-generation EGFR-TKIs (gefitinib or erlotinib)^12,13^.

A pivotal challenge is to understand how major driver mutations affect disease-related molecular networks in the context of lung cancer progression, malignancy and outcome and/or resistance regarding TKI therapies^14^.

Recent advances in high-accuracy mass spectrometry (MS) have made proteomics more compatible with shotgun sequencing and quantitative analysis of disease-related proteins expressed in clinical specimens^14^. Proteomic expression data obtained from such analyses can be used to extract key disease-related proteins and identify novel biomarkers and therapeutic targets^15,16^. A laser microdissection (LMD) technique enables the collection of target cells of a certain type from sections of formalin-fixed paraffin-embedded (FFPE) cancer tissue. We used label-free spectral counting and identification-based semi-quantitative shotgun proteomic analysis of microdissected target cancerous cells of a certain type that characterised lung adenocarcinoma^17–19^.

We recently identified the key protein modules that characterise small-cell lung carcinoma and large-cell neuroendocrine lung carcinoma in systematic network analysis of clinical tissue proteome datasets by weighted gene co-expression network analysis (WGCNA)^20^. WGCNA is an extensively applied unsupervised gene clustering method that is based on the correlation network of gene expression^21–25^. We applied the WGCNA pipeline as well as analysis of variance (ANOVA) to identify the key protein modules and networks affected by the *EGFR* mutations L858R and Ex19del in patients with lung adenocarcinoma.

The main aim of this study was to understand how major driver mutations related to *EGFR* (L858R and/or Ex19del) affect downstream molecular networks and pathways, which would reflect disease nature and treatment outcomes in patients with lung adenocarcinoma (most abundant among NSCLC subtypes) who harbour these *EGFR* mutations. To our knowledge, such a study has not been conducted previously.

## Results

### Proteome datasets of lung adenocarcinoma

MS-based proteomic analysis was conducted for 36 FFPE tissue specimens of lung adenocarcinoma (35 involved the acinar subtype and one involved the papillary subtype). These specimens were selected for their preserved condition, tumour area and well-clarified pathological diagnosis and *EGFR* mutation status (L858R mutation, nine specimens; Ex19del mutation, nine specimens; no Ex19del or L858R mutation, 18 specimens) (Table 1). Pre-surgical treatment was not performed in any of the cases. A total of 3,355 proteins were identified, and of these, about 85% were expressed commonly in the cancer cells of lung adenocarcinoma involving the three mutation statuses. The proportion of proteins unique to each mutation type was less than 0.5%, whereas the proportion of proteins expressed in only no *EGFR* mutation cases was about 5%.

**Table 1:** Clinicopathological information of the 36 patients.

Sample no	Gender	Age	Histological type	Subtype	Surgical method	Tumor size on CT (mm)	Pathological max. size (mm)	Pathological stage	Clinical stage	*EGFR* mutation positive/negative^a^	
Patient 1	F	58	AD	Acinar	Radical lobectomy	18	20	pIA	cIA	Positive	Ex19 E746-A750 del
Patient 2	F	75	AD	Acinar	Radical lobectomy	46	40	pIB	cIB	Positive	Ex19 E746-A750 del
Patient 3	F	72	AD	Acinar	Radical lobectomy	52	39	pIB	cIIA	Positive	Ex19 E746-A750 del
Patient 4	F	72	AD	Acinar	Radical lobectomy	13	20	pIA	cIA	Positive	Ex19 E746-A750 del
Patient 5	M	74	AD	Acinar	Radical lobectomy	18	17	pIA	cIA	Positive	Ex19 E746-A750 del
Patient 6	M	71	AD	Acinar	Radical lobectomy	26	51	pIV	cIV	Positive	Ex19 E746-A750 del
Patient 7	F	71	AD	Acinar	Radical lobectomy	27	28	pIA	cIA	Positive	Ex19 E746-A750 del
Patient 8	F	40	AD	Acinar	Radical lobectomy	26	14	pIIB	cIA	Positive	Ex19 E746-A750 del
Patient 9	F	70	AD	Acinar	Radical lobectomy	24	22	pIA	cIA	Positive	Ex19 E746-A750 del
Average ± SD	M(22.2%) / F (77.8%)	67 ± 10.6				27.8 ± 12.2	27.9 ± 11.9				
Patient 10	M	72	AD	Acinar	Limited resection	18	7	pIB	cIA	Positive	L858R
Patient 11	F	76	AD	Acinar	Radical lobectomy	43	25	pIA	cIB	Positive	L858R
Patient 12	M	71	AD	Acinar	Radical lobectomy	42	37	pIIIA	cIB	Positive	L858R
Patient 13	M	73	AD	Acinar	Radical lobectomy	46	30	pIA	cIB	Positive	L858R
Patient 14	F	64	AD	Acinar	Radical lobectomy	26	18	pIIA	cIA	Positive	L858R
Patient 15	M	64	AD	Acinar	Radical lobectomy	27	30	pIB	cIA	Positive	L858R
Patient 16	F	69	AD	Acinar	Radical lobectomy	45	32	pIV	cIV	Positive	L858R
Patient 17	F	76	AD	Acinar	Limited resection	22	15	pX	cIA	Positive	L858R
Patient 18	M	73	AD	Pap	Radical lobectomy	33	35	pIB	cIB	Positive	L858R
Average ± SD		70.9 ± 4.2				33.6 ± 10.1	25.4 ± 9.5				
Patient 19	M	69	AD	Acinar	Limited resection	15	12	pX	cIA	Negative	
Patient 20	F	80	AD	Acinar	Radical lobectomy	54	45	pIB	cIIA	Negative	
Patient 21	M	73	AD	Acinar	Radical lobectomy	42	30	pIIIA	cIB	Negative	
Patient 22	M	72	AD	Acinar	Radical lobectomy	36	22	pIA	cIB	Negative	
Patient 23	M	63	AD	Acinar	Limited resection	14	11	pX	cIA	Negative	
Patient 24	M	66	AD	Acinar	Limited resection	18	9	pX	cIA	Negative	
Patient 25	F	69	AD	Acinar	Limited resection	7	8	pX	cIA	Negative	
Patient 26	M	55	AD	Acinar	Limited resection	12	20	pIV	cIV	Negative	
Patient 27	M	82	AD	Acinar	Limited resection	25	15	pX	cIA	Negative	
Patient 28	F	70	AD	Acinar	Limited resection	45	45	pX	cIIB	Negative	
Patient 29	M	74	AD	Acinar	Limited resection	18	15	pX	cIA	Negative	
Patient 30	F	62	AD	Acinar	Radical lobectomy	19	22	pIA	cIA	Negative	
Patient 31	M	65	AD	Acinar	Radical lobectomy	31	30	pIA	cIB	Negative	
Patient 32	M	68	AD	Acinar	Limited resection	26	23	pIA	cIA	Negative	
Patient 33	M	63	AD	Acinar	Limited resection	34	27	pIA	cIB	Negative	
Patient 34	M	60	AD	Acinar	Radical lobectomy	28	21	pIA	cIA	Negative	
Patient 35	F	22	AD	Acinar	Radical lobectomy	17	20	pIIIA	cIA	Negative	
Patient 36	M	70	AD	Acinar	Radical lobectomy	28	25	pIA	cIA	Negative	
Average ± SD		65.7 ± 12.4				26.1 ± 12.2	22.2 ± 10.3				
Group comparison											
*p value*_anova		0.517				0.333	0.439				

a, negative means no L858R/Ex19del mutation.

### Identification of key protein modules by WGCNA

**Figure 1:**
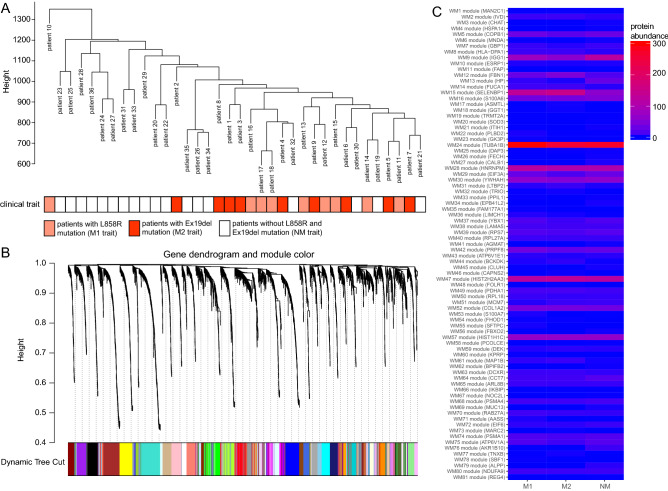
Gene modules identified by weighted gene co-expression network analysis (WGCNA). (**A**) Patient clustering according to protein abundance with the *EGFR* mutation profiles. The red, orange and white cells below the patients indicate the *EGFR* mutation types, i.e., Ex19del mutation, L858R mutation and no *EGFR* mutation, respectively. (**B**) Gene dendrogram obtained by clustering dissimilarity according to topological overlap with the corresponding module. The coloured rows correspond with the 81 modules identified by dissimilarity according to topological overlap. (**C**) Heatmap for the proteome abundance of eigen proteins in the 81 protein modules by WGCNA. The rows and columns are the protein modules and *EGFR* mutation types, respectively. The red and blue colours indicate high and low protein abundances, respectively, of an eigen protein in a protein module. M1, M2 and NM indicate patients with the L858R mutation, those with the Ex19del mutation and those without *EGFR* mutations. The names of the eigen proteins in the protein modules are indicated in parentheses.

The hierarchical clustering of patients according to protein abundance showed limited correlation with the proteome landscape and the *EGFR* mutation type in lung adenocarcinoma (Fig. 1A). We constructed a weighted gene co-expression network and clustered all the identified proteins, and we found 81 protein modules (Fig. 1B,C), which were robustly appeared in the module stability analysis (Supplementary Fig. S1). In the WGCNA, a soft threshold power of 10 was selected to define the adjacency matrix according to the criteria of approximate scale-free topology, with a minimum module size of 5 and a module detection sensitivity *deepSplit* of 4. The clinical traits for patients were set according to the *EGFR* mutation status, with M1, M2 and NM traits corresponding to L858R mutation, Ex19del mutation and no Ex19del/L858R mutation, respectively. The correlations between resultant modules and clinical traits were determined to identify protein modules whose expressions were upregulated or downregulated in L858del, Ex19del or no Ex19del/L858R mutation samples. We identified few modules that showed moderate correlations with clinical traits (|r|> 0.5) (Supplementary Fig. S2).

Among the 81 modules, only the WM44 module was significant with regard to the *EGFR* Ex19del mutation status (r = 0.51, *p* < 0.05). However, several WGCNA modules were suggested to be not significant but relatively characteristic to the three clinical traits (Supplementary Fig. S2). The WM23 module was characteristic to the M1 trait (r = 0.36, *p* < 0.05). The WM12 and WM15 modules showed positive correlations with the M2 trait (r = 0.44, *p* < 0.05 and r = 0.46, *p* < 0.05, respectively) and negative correlations with the NM trait (r =  − 0.41, *p* < 0.05 and r =  − 0.56, *p* < 0.05, respectively). The WM9, WM21, WM39 and WM64 modules were moderately characteristic to the NM trait.

### Statistical analysis of the protein interaction network associated with protein identified by ANOVA

WGCNA is a powerful computational framework to identify co-expression of protein modules. However, traditional trait analysis involving the correlations between eigen components of WGCNA modules and clinical traits might overlook important modules for investigating differential disease mechanisms. ANOVA can identify individual proteins with significant differences in proteome abundance among different patient groups, whereas it cannot identify co-expressed protein modules that might have synergistic and functional protein groups.

**Figure 2:**
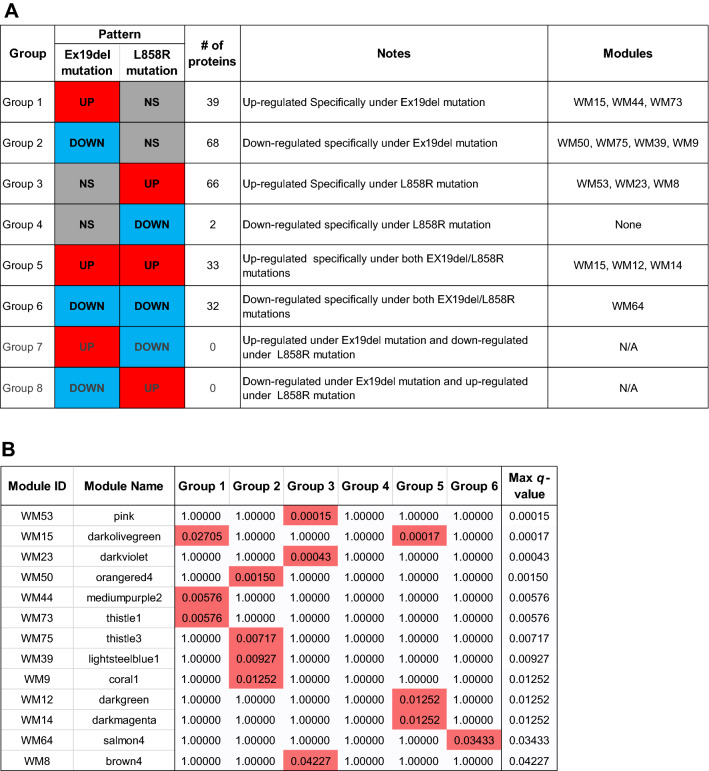
Overlapping proteins from analysis of variance (ANOVA) and those from weighted gene co-expression network analysis (WGCNA). (**A**) ANOVA analysis results. Each row represents results for each protein group (expression patterns, the number of proteins and list of proteins in the group). The red and blue cells in the ‘Ex19del mutation’ and ‘L858R mutation’ columns indicate that the proteins in the group are significantly upregulated and downregulated, respectively, in samples with the mutations (*p* value by ANOVA < 0.05 and adjusted *p* value by the post-hoc pairwise *t* test < 0.05), whereas grey cells in the columns indicate that the proteins in the group are not upregulated/downregulated in samples with the mutations. The fourth column shows the number of proteins in each protein group. The fifth column provides notes for each protein group. The WGCNA modules with significant overlap with each protein group are listed in the sixth column (‘Modules’ column). (**B**) Overlap in proteins between the groups by ANOVA and the modules by WGCNA. Each row in the embedded table represents overlap analysis results for each module. The first and second columns in the table represent module ID and colour name of the module. The third, fourth, fifth, sixth, seventh and eighth columns indicate the *q* values for overlap in proteins between a module by WGCNA and six protein groups by ANOVA (Benjamini–Hochberg correction is performed on the *p* values of the overlap analysis to calculate the *q*-values). In the six columns, significant *q*-values are highlighted in red. The ninth column represents the value of the most significant *q*-value (max *q*-value) in each module. The 13 modules with max *q*-values < 0.05 are listed in order.

Therefore, we conducted ANOVA-based screening of WGCNA modules to further identify key protein modules in order to investigate the differential disease mechanisms associated with the different *EGFR* mutations. ANOVA identified 240 differentially expressed proteins. These proteins were classified into eight groups according to their expression patterns (namely, the combination of mutation types and their directions; *p* value by ANOVA < 0.05 and adjusted *p* value by the post-hoc pairwise *t* test < 0.05) (Fig. 2A). Among the eight groups, six groups had at least two differentially expressed proteins. The protein groups involved several key proteins and pathways that could be useful to investigate the differential disease mechanisms under different *EGFR* mutations (Supplementary Fig. S3). The overlaps between the WGCNA-derived protein modules and ANOVA-based significantly expressed proteins were then assessed using the over-representation test. We identified 13 important WGCNA modules that showed significant overlap (maximum *q*-value among the groups < 0.05) with protein groups from ANOVA (Fig. 2B). These 13 modules included a total of 364 proteins.

### Functional enrichment analysis of selected WGCNA modules obtained by ANOVA-based screening

To characterise the key modules, we analysed the biological connectivity among the proteins in each module by mapping the module proteins in the human protein–protein interaction (PPI) network and among the biological pathways by pathway enrichment analysis (Supplementary Material, Fig. 3, 4 and 5).

#### WGCNA modules associated with the Ex19del mutation

Three (WM15, WM44 and WM73) and four (WM9, WM39, WM50 and WM75) WGCNA modules significantly overlapped with ANOVA-group 1 and group 2, which included proteins significantly upregulated and downregulated under the Ex19del mutation, respectively (Supplementary Material).

Although there was only one protein interaction among proteins in the WM15 module, the enriched pathways of the WM15 module involved SUMOylation of intracellular receptors and negative regulation of the activity of TFAP2 (AP-2) family transcription factors. The SUMO-conjugating enzyme UBE2I (UBE9) in the module is associated with these two pathways. Li et al.^26^ performed an *in-vivo* experiment and demonstrated that upregulated UBC9 enhances migration and invasion of lung cancer cells. Han et al.^27^ reported that, together with the upregulation of SUMO, the UBC9 genotype enhances the sensitivity of irinotecan-based chemotherapy against NSCLC. Interestingly, the enriched pathways of WM15 involve pathways associated with neurological disorders, and monoamine oxidase A (MAOA), a mitochondrial enzyme, is related to these pathways. Defective MAOA is associated with Brunner syndrome and the norepinephrine neurotransmitter release cycle. The expression of MAOA has been shown to increase in various cancers, including prostate cancer and glioma, although the biological role of MAOA in cancer progression is unknown. Recently, Liu et al.^28^ reported that the protein abundance and gene expression levels of MAOA were higher in NSCLC tissues than in non-cancerous lung tissues. Furthermore, they suggested that MAOA might promote NSCLC by modulating epithelial-to-mesenchymal transition (EMT), as the expression levels of MAOA were negatively correlated with those of E-cadherin and positively correlated with those of N-cadherin, Slug and Twist in NSCLC. Additionally, Liao et al.^29^ performed a study involving prostate cancer and suggested that MAOA expression promotes cancer development and that inhibition of MAOA in the epithelial tissue is a useful treatment for adenocarcinoma.

The WM44 module was moderately correlated with the M2 trait, even in the WGCNA itself. The enriched pathways of WM44 involved cAMP-responsive element-binding protein (CREB) phosphorylation, including CREB phosphorylation through RAS activation, ribosomal S6 kinase (RSK) activation and extracellular signal-regulated kinase (ERK)/mitogen-activated protein kinase (MAPK) targeting. Among the proteins in this module, RPS6KA1 (RSK1) is associated with the pathways and phosphorylates members of the MAPK signalling pathway. Lara et al.^30^ demonstrated that RSK1 inhibits cell migration of NSCLC in siRNA analysis and suggested that RSK1 is a potential negative regulator of metastasis in lung cancer.

Yes-associated protein 1 (YAP1), a member protein of the WM73 module, is a critical downstream regulatory target in the Hippo signalling pathway, which is involved in development, growth, repair and homeostasis and especially plays a pivotal role in the development and progression of various cancers^31^. The enriched pathways of the WM73 module involve YAP1- and WW domain-containing transcription regulator protein 1 (WWTR1) (alternative name, transcriptional coactivator with PDZ-binding motif (TAZ)) (YAP/TAZ)-stimulated gene expression, and RUNX3 regulates YAP1-mediated transcription and dominantly indicates the participation of the Hippo signalling pathway. When the Hippo pathway is off, dephosphorylated YAP/TAZ accumulates and translocates into the nucleus to bind transcription factors (TEA domain family members [TEAD]), which transcribe genes involved in cell proliferation and anti-apoptosis function. Amphiregulin (AREG), a ligand for EGFR, which is one of the transcriptional targets of YAP, is known to cause resistance to chemotherapy and receptor tyrosine inhibitors, such as gefitinib, in patients with NSCLC^32,33^.

**Figure 3:**
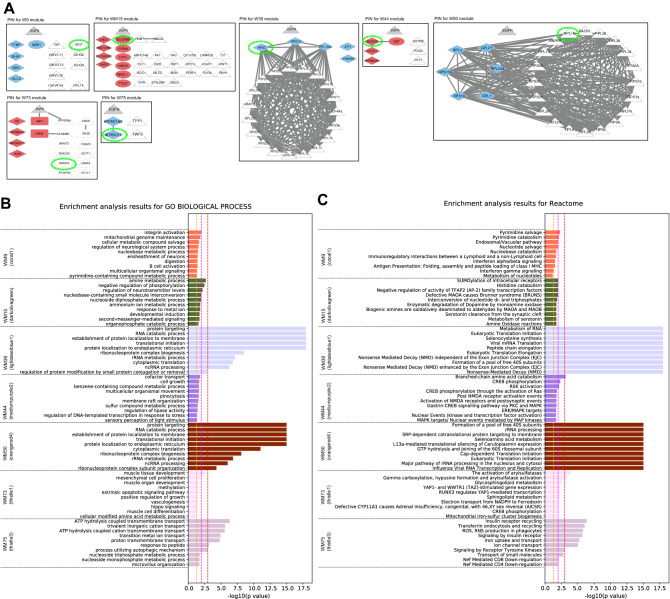
Analysis results for three protein modules (WM15, WM44 and WM73) and four protein modules (WM9, WM39, WM50 and WM75) that overlap with proteins upregulated and downregulated under the Ex19del mutation, respectively. (**A**) Protein interaction networks for the seven protein modules. Diamond nodes in red and blue represent the proteins upregulated and downregulated under the Ex19del mutation, respectively. Rectangle nodes in red represent proteins upregulated under the L858R mutation. Circle nodes in red and blue represent proteins upregulated and downregulated under both *EGFR* mutations, respectively. The eigen protein in each module is denoted by a yellow-green dot circle. Triangle nodes in white represent proteins in each protein module. Triangle nodes in grey represent the EGFR protein. B and C. Pathway enrichment analysis using Go Biological Process (**B**) and Reactome pathway databases (**C**) for the seven protein modules. The vertical axis shows the pathway names, and the bars on the horizontal axis represent the − log10 (*p* value) of the corresponding pathway. The different colours of the bars are in accordance with the corresponding modules. Dashed lines in orange, magenta and red indicate *p* values < 0.05, < 0.01 and < 0.001, respectively.

Among the WM9, WM39, WM50 and WM75 modules, the WM39 and WM50 modules showed dense PPI subnetworks where most of the proteins interacted with each other (Fig. 3A), and both modules were enriched with proteins related to translation initiation pathways (Fig. 3B,C). Especially, proteins in the subnetwork of the WM39 module were involved in the nonsense-mediated decay (NMD) pathway that is a key component to maintain the quality and quantity of transcripts through elimination of mRNA with a premature stop codon. Tumours exploit the NMD pathway to optimise gene expression for their survival, i.e., tumours downregulate the expression of tumour suppressor genes by fine-tuning the NMD pathway^34^. The greatly downregulated proteins RPS13, PRL10A and RPS7 under the Ex19del mutation were involved in the subnetwork of the WM39 module and had important roles in the disease mechanisms associated with this mutation status. Upregulation of RPS13 is associated with multi-drug resistance in gastric cancers^35^. Recently, Shi et al.^36^ reported that upregulation and downregulation of RPL10 respectively increased and decreased viability, migration and invasion of tumour cells in epithelial ovarian cancer. Furthermore, downregulation of RPS7 has been shown to promote the migration of tumour cells in ovarian^37^ and prostate^38^ cancers through representative PI3K/AKT and MAPK cancer signalling pathways and EMT, respectively.

#### WGCNA modules associated with the EGFR L858R mutation

**Figure 4:**
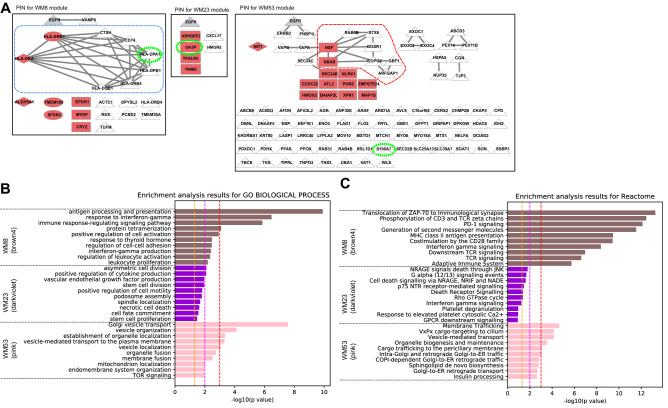
Analysis results for three protein modules (WM8, WM23 and WM53) that overlap with proteins upregulated under the L858R mutation. (**A**) Protein interaction networks for the three protein modules. (**B**) Pathway enrichment analysis using Go Biological Process. (**C**) Pathway enrichment analysis using Reactome pathway databases. The legend for each panel is the same as that for Fig. 3.

The WM8, WM23 and WM53 modules significantly overlapped with ANOVA-group 3, which included proteins specifically upregulated under the L858R mutation (Supplementary Material). PPI subnetworks were found in the WM53 and WM8 modules, which reflected specific disease mechanisms associated with the L858R mutation (Fig. 4A). As shown in Fig. 4B,C, the WM53 module was significantly enriched with proteins from the Golgi to endoplasmic reticulum (ER) retrograde traffic system (ARFGAP1, COPG2, GBF1, GOSR1, NBAS, NSF, RAB6B and STX6). The enriched pathways of the WM23 module involved the p75 neurotrophin receptor (p75^NTR^)-interacting protein (NRAGE) signalling death through JNK and cell death signalling via NRAGE, neurotrophin receptor interacting factor (NRIF) and p75^NTR^-associated cell death executor (NADE). The WM8 module was associated with immune pathways, including PD-1 signalling, interferon-gamma signalling and regulation of leukocyte activation (CD74, CTSH, HLA-DPA1, HLA-DPB1, HLA-DQB1, HLA-DRA, HLA-DRB1 and HLA-DRB5) (Fig. 4B,C).

#### WGCNA modules associated with both Ex19del and L858R mutations

**Figure 5:**
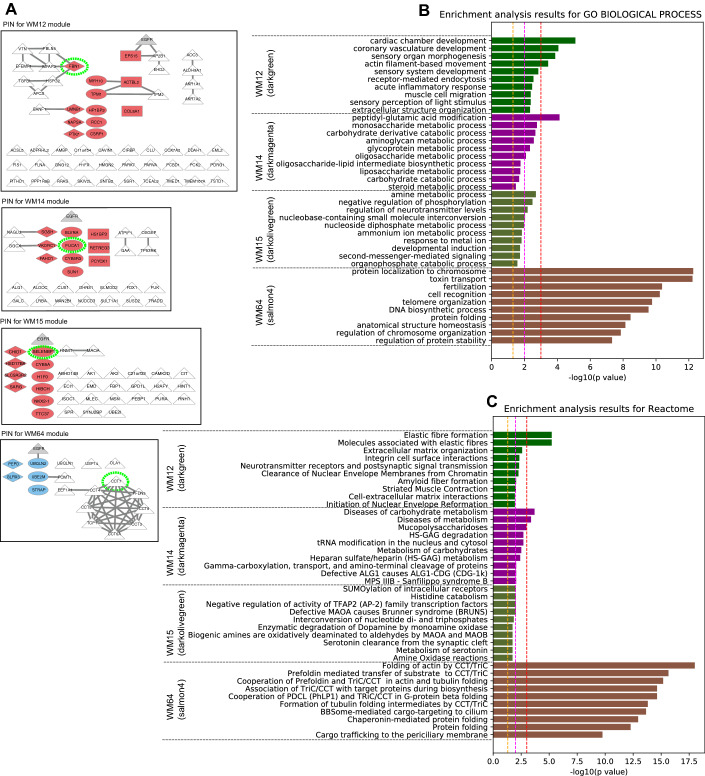
Analysis results for four protein modules (WM12, WM14, WM15 and WM64) that overlap with proteins upregulated or downregulated under both L858R and Ex19del mutations. (**A**) Protein interaction networks for the four protein modules. (**B**) Pathway enrichment analysis using Go Biological Process. (**C**) Pathway enrichment analysis using Reactome pathway databases. The legend for each panel is the same as that for Fig. 3.

The WM15, WM12, WM14 and WM64 modules significantly overlapped with ANOVA-group 5 and group 6, which included proteins that were upregulated and downregulated, respectively, in the same direction in both Ex19del and L858R mutations (Supplementary Material). Among the four modules, the WM12 and WM64 modules showed PPI subnetworks (Fig. 5A). The PPI subnetwork in the WM64 module was tightly associated with the proteins in cytosolic chaperonin TRiC/CCT pathways (Fig. 5B,C). Interestingly, the proteins CCT5 and EEF1 in the subnetwork are known to be potential tumour-associated antigens that could be useful in the development of a diagnostic biomarker for NSCLC^39^.

### Comparative analysis of causal network inactivation or activation predicted by Ingenuity Pathway Analysis

**Figure 6:**
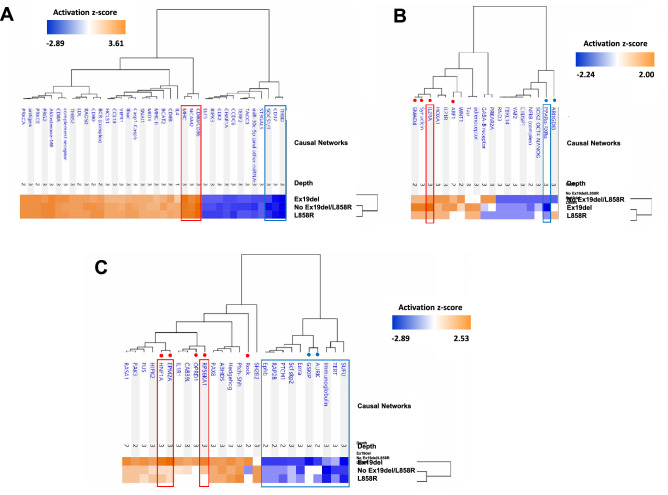
Comparative analysis results of causal networks predicted for three protein modules (WM15, WM44 and WM73) that overlap with proteins upregulated under the Ex19del mutation. (**A**) WM15 (|z-score|> 2.0), (**B**) WM44 (|z-score|> 1.2) and (**C**) WM73 (|z-score|> 1.5). The top causal networks with high activation z-scores are compared and shown with their hierarchical clustering together with the mutation status using the Ingenuity Pathway Analysis comparison. Orange and blue indicate upregulation and downregulation, respectively. Causal networks surrounded by red/blue frames are activated/inhibited according to the definition |z-value|≥ 2.0. Causal networks dotted in red/blue are likely to be upregulated/downregulated differentially among the three mutation types.

**Figure 7:**
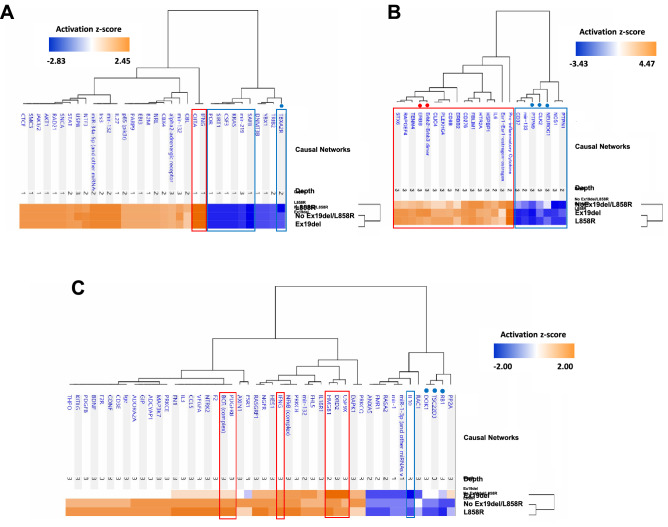
Comparative analysis results of causal networks predicted for three protein modules (WM8, WM53 and WM23) that overlap with proteins upregulated under the L858R mutation. (**A**) WM8 (|z-score|> 1.2), (**B**) WM53 (|z-score|> 2.6) and (**C**) WM23 (|z-score|> 1.3). The legend is the same as that for Fig. 6.

The primary reasons for performing ANOVA-based screening of WGCNA modules were identification of clinically significant modules and their key networks/upstream regulators and further investigation of the disease mechanisms affected differentially under the different driver *EGFR* mutations in lung adenocarcinoma. We conducted an analysis of upstream regulators and causal networks for the 13 modules by using Ingenuity Pathway Analysis (IPA, https://www.ingenuity.com) software^40^. Comparative analysis of predicted causal networks was then performed especially for the three modules significantly associated with the Ex19del mutation (WM15, WM44 and WM73) and the three modules significantly associated with the L858R mutation (WM53, WM23 and WM8) (Figs. 6 and 7).

All causal networks for the WM15 module were suggested to be quite similar in their extent of activation or inhibition in all clinical traits. CD80/CD86 (group) (z-score > 3.0), major histocompatibility complex (MHC) (complex) (z-score > 3.2) and NCAM2 (z-score > 2.8) were highly activated causal networks, and THBD (transmembrane receptor) (z-score <  − 2.8), CD37 (z-score <  − 2.4) and SOCS1/3 (z-score <  − 2.0) were highly inhibited causal networks (Fig. 6A).

CD80/CD86 (known as B7, B7-1/B7-2 or Cd152I) is an immune-checkpoint protein from the B7 family, which has seven members, including B7-DC (PD-L2), B7-H1 (PD-L1) and B7-H2 (ICOSL), and it binds CD28 and CTLA-4. Costimulatory pathways of the B7/CD28 family provide positive and/or negative secondary signals to antigen-experienced effector T cells. Together with the activation of MHC complex networks, costimulatory signal pathways upregulating the expression of inhibitory B7 molecules, which promote tumour immune evasion, appear to be activated^41,42^. In this context, CD37 (TSPAN26), which is highly inhibited, is known to play important roles in T cell–B cell interactions (a balance between immune response and tolerance), and it is widely expressed in normal and malignant mature B cells. CD37 has been reported to directly mediate transduction associated with survival and apoptosis^43^, and recently, it has been suggested to be a biomarker for PD-1 blockade in diffuse large B-cell lymphoma^44^. THBD (also known as thrombomodulin, fetomodulin, and CD141) is not only a thrombin receptor but also an oncodevelopmental antigen, which is considered to modulate cancer cell behaviour related to anticoagulant activity^45^.

Among causal networks predicted for the WM44 module, those upregulated and differentially associated with the Ex19del mutation were interleukin 2 receptor alpha (IL2RA) (z-score = 2.0), ADP-ribosylation factor 1 (ARF1) (z-score = 1.41) and mothers against decapentaplegic homolog 4 (SMAD4) (z-score = 1.73), whereas the highly inhibited network was peroxisome proliferator-activated receptor alpha-retinoid X receptor alpha (PPARα-RXRα) (z-score =  − 2.24) (Fig. 6B).

IL2RA is a cytokine receptor of the IL2R family, which is expressed in many types of cancers, including leukaemia, lymphoma, lung cancer, breast cancer, head and neck cancer and prostate cancer. It participates in various pathways, including inducible costimulator (iCOS)-iCOS ligand signalling in T-helper cells, IL-2 signalling and the PD-1/PD-L1 cancer immunotherapy pathway. Its high expression in tumours is correlated with a poor prognosis^46^. ARF1 is a small G protein that regulates reorganisation of the actin cytoskeleton and plays a major role in protein trafficking in cells. Overexpression of ARF1 has been reported to result in cell proliferation and migration through the PI3K signal pathway in ovarian cancer^47^ and proliferation of breast cancer cells through regulation of the retinoblastoma protein^48^. SMAD4 participates in SMAD4-dependent TGF-β signalling, which is common during tumour development and progression, and it might act as a tumour suppressor by inhibiting cell proliferation. However, SMAD4 also promotes cell motility and the EMT process in most epithelial cells^49^. PPARα-RXRα pathways play key negative roles regarding inflammation.

With regard to the WM73 module, the highly upregulated causal networks associated with the Ex19del mutation were Rock (group) (z-score = 1.89), EPM2A (z-score = 2.53), HNF1A (z-score = 2.33), RPS6KA1 (z-score = 2.11) and OPRD1 (δ-opioid receptor) (z-score = 1.67), and the inhibited networks were GSKIP (z-score =  − 2.11) and aurora kinase (AURK) (z-score =  − 1.73) (Fig. 6C).

Rock (group) (Rho-associated coiled‑coil containing protein kinase 1 and 2 [ROCK1 and 2]) is highly important in oncogenesis, and it promotes the invasive and metastatic growth of a variety of human cancers^50^. ROCK1 and ROCK2 have been reported to play crucial roles in cell cycle progression and tumorigenesis^51^ and to be required for NSCLC anchorage-independent growth and invasion^50^. Thus, Rock has been a promising therapeutic target for NSCLC. GSKIP is a glycogen synthase kinase-3 (GSK3) β-interacting protein, which is involved as a negative regulator of GSK3-β in the Wnt signalling pathway^52^. Interestingly, the AURK (A, B and C) network was mostly downregulated in lung adenocarcinoma patients with the Ex19del mutation. It is known that residual disease and acquired resistance in response to EGFR TKIs requires aurora kinase A (AURKA) activity^53^.

The top canonical pathways predicted for the WM8 module among all the clinical traits were the antigen presentation pathway (*p* = 2.00 × 10^−17^), allograft rejection signalling pathway (*p* = 2.51 × 10^−12^), OX40 signalling pathway (*p* = 3.16 × 10^−12^; z-score =  − 2), PD-1/PD-L1 cancer immunotherapy pathway (*p* = 1.26 × 10^−11^; z-score =  − 2) and T helper 1 pathway (*p* = 3.16 × 10^−11^; z-score = 2). In all mutation types, CIITA and IFNG networks were activated (z-scores = 2.45) but scaffold attachment factor B (SAFB), KRAS, CSF3, SIRT1 and POR networks were downregulated (z-scores ranging from − 1.73 to − 2.00) (Fig. 7A).

CIITA (MHC class II transactivator) acts as a regulator of MHC class II gene transcription (‘master control factor’), which is involved in antigen processing and presentation pathways. IFNG encodes interferon-gamma and is the most powerful MHC inducer that triggers both MHC-I and MHC-II expressions. SAFB is a nuclear matrix protein that binds to the scaffold or the matrix attachment region (S/MAR) in DNA^54^, and its downregulation has been reported to be significantly associated with poor survival among patients with colorectal cancer (CRC)^55^.

No causal networks differentially upregulated or downregulated under the L858R mutation were identified for the WM53 module. Networks related to each other, which included Erbb2-Erbb3 dimer (z-score > 2.5), ERBB (z-score > 2.8) and ERBB2 (z-score > 1.9), appeared to be associated more differentially with the Ex19del mutation (Fig. 7B). CDC like kinase 2 (CLK2) was highly inhibited under the L858R mutation (z-score =  − 2.6). Loss of CLK2 in luminal breast cancer cells has been reported to result in the upregulation of EMT-related genes and a switch to the mesenchymal splice variants of several genes^56^.

For the WM23 module, under the L858R mutation, no networks were differentially upregulated, but the RB1 and DOK1 networks were differentially downregulated (Fig. 7C). RB1 is a well-known tumour suppressor protein (pRb), and DOK1 (docking protein 1) is also a tumour suppressor, which shows repressed expression in many human tumours owing to hypermethylation of its promotor region^57^.

## Discussion

Understanding disease-related molecular mechanisms and profiles in lung adenocarcinoma affected differently depending on the type of *EGFR* gatekeeper mutation would be greatly useful in deciding treatment strategies that benefit the outcomes of individual patients. Our new analytical workflow combining ANOVA and WGCNA identified several protein modules and networks that were potentially associated with disease mechanisms driven by distinct *EGFR* mutations.

The pathways of SUMOylation and transitions between epithelial and mesenchymal states (EMT/MET) were centrally associated with the WM15 module. SUMOylation is a reversible post-translational modification, which is crucial to regulate several key disease mechanisms including DNA damage repairing system, immune responses, carcinogenesis, cell cycle progression, and apoptosis. SUMOylation regulates the transcription activity of the AP-2 family, which is one of the transcription factors controlling transitions between epithelial and mesenchymal states (EMT/MET)^58^. The top IPA network constructed from member proteins was associated with cell-to-cell signalling and interaction, small molecule biochemistry and cancer, where both SENENBP1 and MAOA were upregulated. The eigen protein selenium-binding protein 1 (SELENBP1) (also known as SBP1 or hsP56), a member of the selenoprotein family, mediates intercellular transport of selenium and is known to interact with various other proteins including TGF-β, HIF-1α, von Hippel-Lindau protein-interacting deubiquitinating enzyme 1, TWIST1 (a critical regulator of EMT) and tumour protein p53. It has been reported that downregulation of SELENBP1 is often associated with tumour progression in various epithelial cancers, including lung cancer, and with poor clinical outcomes^59^. SELENBP1 is currently considered a tumour suppressor that regulates cell proliferation, senescence, migration and apoptosis. Interestingly, SELENBP1 demonstrated a clear bimodality in its protein abundance distribution across all 36 samples (Supplementary Material), suggesting two modes involving high and low expressions of the protein, which appear to depend on the *EGFR* mutation status (positive and negative). Our observation might suggest a bimodal response of the SELENBP1 gene regulatory system, which delineates the survival of two cancer cell populations with different origins^60^.

The activation of the ERK/MAPK pathway via phosphorylation of the members, which is a major downstream via cascades of signals by EGFR activation, is likely associated with the WM44 module. The WM73 module evidently suggested participation in the Hippo signalling pathway that might interact with the ERBB/EGFR signalling pathway, forming a positive feed‐forward loop to drive cancer development and progression.

The WM23, WM53 and WM8 modules appeared to be involved in various pathways related to cancer cell survival and death, such as the Golgi to ER retrograde traffic system; NRAGE signalling death through JNK and cell death signalling via NRAGE, NRIF and NADE; and immune pathways including PD-1 signalling, MHC class II antigen presentation and interferon-gamma signalling. The Golgi apparatus has been recognised for its central role in tumour cell survival, which is regulated by ARF1 activated by ARF guanine nucleotide exchange factors.

More than 280 causal networks, which were predicted to be upregulated or downregulated with |activation z-scores|> 1.0, were elucidated for the WM15, WM44 and WM73 modules and for the WM53, WM23 and WM8 modules, using IPA comparative analysis. Hierarchical clustering by the mutation status suggested that causal networks predicted for the WM15, WM44 and WM23 modules were associated with the Ex19del mutation and that those predicted for only the WM8 module were associated with the L858R mutation (Figs. 6 and 7). Thus, more causal networks were suggested to be associated with the Ex19del mutation, and they involve the upregulation of Rock (group), RPS6KA1, EPM2A, HNF1A, ARF1, IL2RA, SMAD4, Erbb2-Erbb3 dimer and ERBB and the downregulation of AURK, GSKIP, NEULOG1, CLK2 and PTPN9. On the other hand, under the L858R mutation, causal networks involving RB1, TSC22D3 and DOCK1 were downregulated, although various causal networks involving F2, NTRK2 and VEGFA were moderately upregulated (Fig. 7C). Causal networks predicted for the WM15, WM73 and WM23 modules were clustered as one group with regard to both L858R mutation and no Ex19del/L858R mutation. Such profiles of causal networks in activation might imply that the downstream biological networks are influenced by factors other than the L858R mutation, which have not been identified. Causal networks common to all the mutation types included highly upregulated CD80/CD86 (B7), MHC, CIITA and IFGN and highly downregulated CD37 and SAFB. For most patients in this study, it was interestingly suggested that costimulatory immune-checkpoint pathways by B7/CD28 were activated, whereas those by PD-1/PD-L1 were inhibited. This result seems consisitent with clinical findings that the PD-1/PD-L1 cancer immunotherapy pathway is not activated in early-stage lung adenocarcinomas.

With regard to the therapeutic effects of EGFR-TKIs in *EGFR* mutation-positive NSCLC, PFS was found to differ depending on the mutation subtype (Ex19del or L858R), and PFS tended to be longer in patients with Ex19del than in those with Ex21 L858R, regardless of the EGFR-TKI therapies received (osimertinib and standard TKIs)^12^. Various studies have been attempted to provide explanations for this difference in therapeutic outcomes according to the *EGFR* mutation type. Several hypotheses have been proposed. First, L858R-positive *EGFR* is highly phosphorylated, which might result in a poor prognosis owing to its high cell-proliferative capacity, according to cell-line assessments^61^. Second, L858R-positive NSCLC harbours many miscellaneous compound mutations other than *EGFR* mutation^62^, whereas Ex19del-positive NSCLC is more likely to be relatively pure with regard to the oncogene mutation that drives proliferation and is mainly dependent on the EGFR pathway, which would result in a long PFS with EGFR-TKIs. Our comparative analysis results for causal networks among the *EGFR* mutation types might support the latter hypothesis, because there were no activated or inhibited causal networks particularly related to downstream EGFR signalling or uniquely related to the L858R mutation. However, further investigation is required. At present, EGFR-TKI therapy has clinical efficacy limitations with regard to L858R-positive NSCLC. Therefore, combination therapy involving EGFR-TKIs and other drugs, such as anticancer drugs and angiogenesis inhibitors, is considered to be more effective for L858R-positive NSCLC. Indeed, therapy involving an anti-VEGF antibody (bevacizumab, an angiogenesis inhibitor) after an EGFR-TKI (erlotinib) demonstrated particularly effective outcomes, with an increase in PFS among patients with L858R-positive NSCLC^63^.

In conclusion, we successfully applied WGCNA combined with ANOVA-based protein screening to clinical proteomic datasets from 36 patients with lung adenocarcinoma. Our results could lead to the identification of activated or inactivated disease-related networks that are possibly affected under distinct *EGFR* mutations. Additionally, our findings may help in the development of therapeutic strategies to improve patient outcomes. A further in-depth network-based investigation on the tumorigenesis of lung adenocarcinoma under different *EGFR* mutations will provide clinically important information on proteogenomic landscapes in lung adenocarcinoma.

## Methods

### FFPE tissue specimens and sample preparation

Among 974 patients who underwent surgical lung cancer resection at St. Marianna University Hospital between 2000 and 2014, only 674 (69.3%) had tumours that were histologically confirmed adenocarcinomas. Pathological specimens were reviewed independently by two pathologists (H.N. and M.T.) to confirm that they satisfied the 2015 WHO classification of lung tumours (histological criteria)^64^. FFPE tumour tissue blocks from 36 surgical specimens of lung adenocarcinomas with known *EGFR* mutation statuses were obtained without patient identifiers from St. Marianna University School of Medicine Hospital. Informed consent was obtained from all participating subjects, and the protocol was approved by the institutional review board of St. Marianna University School of Medicine (approval no. 3569) and was conducted in accordance with the Helsinki Declaration. For tissue microdissection, 10-μm-thick sections from the FFPE tumour blocks were cut onto DIRECTOR slides (OncoPlex Diagnostics Inc., Rockville, MD, USA). The sections were de-paraffinised and stained with hematoxylin using standard histological methods prior to dissection. Microdissection was performed using a Leica LMD7 Microdissection Microscope (Leica, Wetzlar, Germany). A total area of 4 mm^2^ with about 15,000 tumour cells was transferred from the FFPE sections via laser dissection directly into the cap of a 200-μL low-binding tube. Proteins were extracted and digested with trypsin using Liquid Tissue MS Protein Prep kits (OncoPlex Diagnostics Inc.) according to the manufacturer’s protocol^65^. Details of the procedures have been described in detail elsewhere^17–19^.

### Liquid chromatography-tandem mass spectrometry-based proteomic analysis

A label-free quantitation approach using spectral counting by liquid chromatography–tandem MS (LC–MS/MS) was adopted for global proteomic analysis. The digested samples (5 μL for a single run) were analysed in triplicate by LC–MS/MS using a reverse-phase LC system interfaced with a Q Exactive Orbitrap mass spectrometer (Thermo-Fisher Scientific, Bremen, Germany) via a nano-ESI device (AMR Inc., Tokyo, Japan). LC–MS/MS analysis has been described in detail previously^19^. The expression levels of identified proteins were assessed by spectral count-based protein quantification. The spectral count is the number of MS/MS spectra assigned to each protein.

### WGCNA

The similarity in protein expression patterns for all protein pairs was calculated according to their pairwise Pearson’s correlation coefficient, i.e., the similarity between proteins i and j was defined as (1 − *r*_*i,j*_)/2, where *r*_*i,j*_ is the Pearson’s correlation coefficient of the protein expression patterns between these two proteins. We performed the network topology analysis for various soft-thresholding powers ranging from 1 to 100 in order to choose an optimal value to balance between independence and mean connectivity. The power had been set to 10 where scale independence score was above 0.90 and the network reaching to scale-free topology. Subsequently, from the resultant scale-free co-expression network, we generated a topological overlap matrix (TOM) that considers topological similarity between a pair of proteins in the network. By using the dissimilarity according to the TOM (1 − TOM), we conducted hierarchical clustering to generate a tree that clustered proteins in its branches. Dynamic tree cutting was used to trim the branches to identify protein modules. A protein module was summarised by the top hub protein (referred to as the eigen protein) with the highest connectivity in the module. In order to identify protein modules associated with clinical traits, we calculated Pearson's correlation coefficients between the eigen proteins and clinical traits. We used the flushclust library in R:Bioconductor to get the hierarchical cluster where complete linkage method was applied. The euclidean distance and topological overlap matrix (TOM) dissimilarity were used as a distance measure for the clustering. The module stability analysis was performed using Fast R function in WGCNA according to the reference^66^. WGCNA was conducted by using a Garuda Platform gadget (The Systems Biology Institute, Tokyo Japan) that implements the WGCNA pipeline based on WGCNA R-package^21^. The codes are available upon request.

### Statistical analysis for differentially expressed proteins

To identify differentially expressed proteins, we conducted one-way ANOVA with a post-hoc statistical test (pairwise *t* test) for three patient groups (patients with the Ex19del mutation, those with the L858R mutation and those without bothf these mutations). In the statistical analysis, we identified proteins significantly upregulated/downregulated in samples with the mutations (*p* value by ANOVA < 0.05 and adjusted *p* value by post-hoc pairwise *t* test < 0.05). We conducted the statistical analysis using anova and lm fuctions in R programming language.

### Protein–protein interaction network construction

In order to construct a protein interaction network (PIN) for a protein module as well as protein groups by ANOVA analysis, we used the STRING (The Search Tool for the Retrieval of Interacting Genes/Proteins) database (version 10.5)^67^. Proteins in a protein module (or those in protein groups by ANOVA analysis) were mapped in the PIN from the STRING database, and a subnetwork involving protein–protein interactions connecting these proteins was extracted. We regarded the subnetwork as a PIN for the protein module (or that for the protein groups by ANOVA analysis).

### Functional enrichment analysis

In enrichment analysis, the focused set of genes underwent the statistical over-representation test (Fisher’s exact test with FDR multiple test correction) on either the Go Biological Process or Reactome pathway database. We used the Web-based Gene SeT AnaLysis Toolkit (WebGestalt)^68^ for the analysis.

### Comparative analysis of the causal networks and pathways predicted by IPA

Canonical pathways, upstream regulators and causal networks were predicted using the IPA software^40^. Protein expression data (quantile-normalised for selected modules) were used as input datasets. Comparative analysis of the predicted causal networks (*p* value < 0.05) was performed to elucidate networks associated with the three clinical traits of Ex19del mutation, L858R mutation and no Ex19del/L858R mutation, where activation and inhibition of a predicted network were defined by z-values > 2.0 and <  − 2.0, respectively, and upregulation and downregulation were defined by z-values > 1.0 and <  − 1.0, respectively.

## Supplementary information

Supplementary file1 (PDF 5063 kb)

## Data Availability

The unfiltered mass spectrometry datasets generated and analysed in this study have been deposited in the PRIDE Archive (https://www.ebi.ac.uk/pride/archive/) via the PRIDE partner repository and jPOST with the dataset identifiers PXD015862 and JPST000687, respectively.
