# Supplementary material for: Disease-related cellular protein networks differentially affected under different EGFR mutations in lung adenocarcinoma
Source: Sci Rep. 2020 Jul 2;10:10881. doi: 10.1038/s41598-020-67894-7 (PMC7331587; doi:10.1038/s41598-020-67894-7)
Supplement: Supplementary file 1 — Supplementary file1 (PDF 5063 kb) [file 41598_2020_67894_MOESM1_ESM.pdf]

Supplementary Information

Disease-related Cellular Protein Networks Differentially Affected under Different *EGFR* Mutations in Lung Adenocarcinoma

Toshihide Nishimura, Haruhiko Nakamura, Ayako Yachie, Takeshi Hase, Kiyonaga Fujii, Hirotaka Koizumu, Saeko Naruki, Masayuki Takagi, Yukiko Matsuoka, Naoki Furuya, Harubumi Kato, Hisashi Saji

Supplementary Figure S1. WGCNA module stability analysis.

The upper panel demonstrates the hierarchical clustering based dendrogram of all proteins using the all samples in the dataset. Branches of the dendrogram associated with modules that are indicated with colored solid blocks in the color row for the full data set. Module assignment results of the 20 resampled dataset where 90% of the patient samples were randomly sampled were shown below the module of the full data set.

Gene dendrogram and module labels from resampled data sets

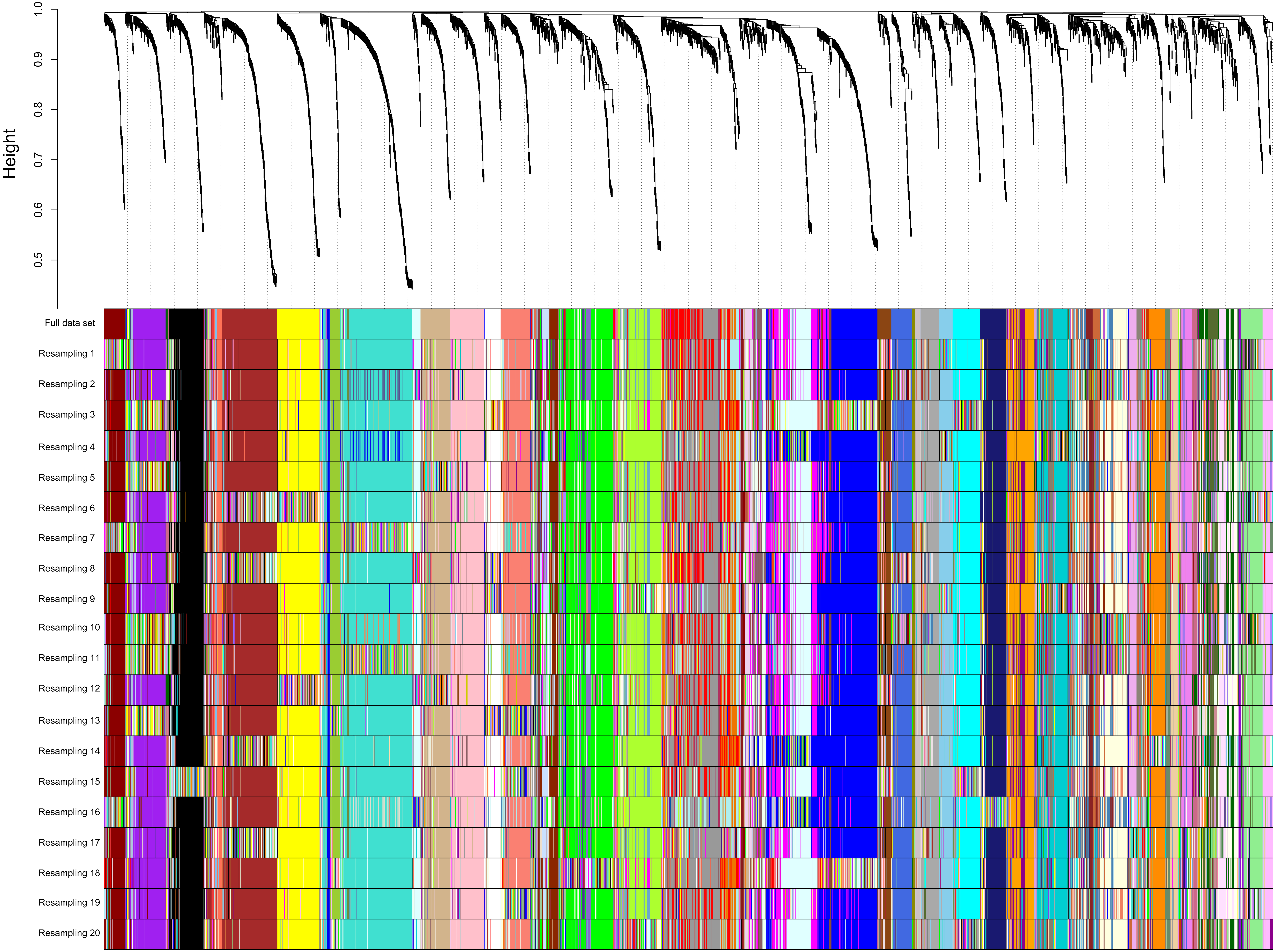

Supplementary Figure S2.

Relationship between module eigen proteins and the L858R and Ex19del mutations in the *EGFR* gene.

Each row in the embedded table represents weighted gene co-expression network analysis results for each module. The first and second columns in the table represent module ID and colour name of the module. The third, fourth and fifth (sixth, seventh and eighth) columns indicate the correlation coefficients (*p*-values of the correlation coefficients) between the corresponding modules and the clinical traits. The ninth column represents the number of proteins in each module. The table is colour-coded by correlation coefficient according to the colour legend on the right side of the figure. The intensity and direction of the correlations are indicated on the right side of the heatmap (red, positive correlation; blue, negative correlation). Significant *p*-values are highlighted in red.

Module ID	Module Name	module trait relationships						# of genes in the module
		trait M1 (correlation)	trait M2 (correlation)	trait NM (correlation)	trait M1 (p-value)	trait M2 (p-value)	trait NM (p-value)	
WM1	antiquewhite4	-0.10	0.09	0.01	0.5586	0.6133	0.9454	14
WM2	bisque4	0.07	0.28	-0.30	0.6948	0.0936	0.0711	23
WM3	black	-0.10	-0.10	0.17	0.5698	0.5754	0.3261	99
WM4	blue	-0.12	-0.12	0.21	0.4707	0.4825	0.2140	159
WM5	blue2	0.14	-0.40	0.23	0.4307	0.0147	0.1733	7
WM6	brown	-0.07	-0.11	0.16	0.6789	0.5305	0.3654	158
WM7	brown2	-0.02	-0.23	0.22	0.8875	0.1760	0.1955	7
WM8	brown4	0.11	0.31	-0.36	0.5230	0.0659	0.0293	23
WM9	coral1	-0.23	-0.32	0.48	0.1687	0.0557	0.0029	15
WM10	coral2	0.09	-0.03	-0.06	0.5832	0.8575	0.7499	12
WM11	cyan	-0.10	-0.09	0.17	0.5652	0.5891	0.3318	80
WM12	darkgreen	0.03	0.44	-0.41	0.8628	0.0074	0.0139	58
WM13	darkgrey	-0.15	-0.19	0.30	0.3676	0.2639	0.0759	51
WM14	darkmagenta	0.15	0.34	-0.42	0.3911	0.0425	0.0103	31
WM15	darkolivegreen	0.19	0.46	-0.56	0.2766	0.0050	0.0004	33
WM16	darkolivegreen4	-0.19	-0.01	0.17	0.2565	0.9693	0.3104	7
WM17	darkorange	-0.10	0.29	-0.17	0.5687	0.0811	0.3212	46
WM18	darkorange2	-0.04	0.26	-0.19	0.8340	0.1262	0.2582	23
WM19	darkred	-0.05	-0.11	0.14	0.7539	0.5280	0.4121	60
WM20	darkseagreen4	-0.05	-0.12	0.14	0.7916	0.4992	0.4145	15
WM21	darkslateblue	-0.26	-0.28	0.47	0.1258	0.1011	0.0042	21
WM22	darkturquoise	0.25	-0.10	-0.13	0.1407	0.5763	0.4377	56
WM23	darkviolet	0.36	-0.19	-0.15	0.0307	0.2674	0.3891	6
WM24	firebrick4	0.13	-0.20	0.07	0.4598	0.2358	0.7047	7
WM25	floralwhite	-0.21	-0.06	0.23	0.2169	0.7401	0.1729	24
WM26	green	-0.11	-0.12	0.20	0.5183	0.4889	0.2433	119
WM27	greenyellow	-0.10	-0.10	0.17	0.5707	0.5728	0.3250	93
WM28	grey60	0.26	0.03	-0.25	0.1201	0.8792	0.1395	68
WM29	honeydew1	0.10	-0.44	0.29	0.5671	0.0076	0.0820	16
WM30	indianred4	-0.06	-0.12	0.16	0.7277	0.4709	0.3528	7
WM31	ivory	-0.01	0.29	-0.24	0.9480	0.0903	0.1615	24
WM32	lavenderblush3	-0.02	0.26	-0.21	0.9067	0.1233	0.2212	16
WM33	lightcoral	-0.07	-0.27	0.29	0.6852	0.1154	0.0842	7
WM34	lightcyan	-0.11	-0.11	0.19	0.5287	0.5322	0.2744	69
WM35	lightcyan1	0.29	-0.09	-0.17	0.0844	0.5859	0.3181	24
WM36	lightgreen	0.26	-0.01	-0.22	0.1189	0.9624	0.1930	66
WM37	lightpink4	0.12	-0.37	0.22	0.4904	0.0247	0.1953	17
WM38	lightsteelblue	0.02	0.05	-0.06	0.9205	0.7782	0.7410	7
WM39	lightsteelblue1	-0.20	-0.27	0.41	0.2504	0.1059	0.0136	25
WM40	lightyellow	-0.16	0.25	-0.08	0.3657	0.1490	0.6508	66
WM41	magenta	-0.14	-0.14	0.24	0.4081	0.4158	0.1511	96
WM42	maroon	0.28	-0.11	-0.14	0.1024	0.5046	0.4151	17
WM43	mediumorchid	-0.05	-0.18	0.20	0.7932	0.2916	0.2527	12
WM44	mediumpurple2	-0.04	0.51	-0.40	0.7986	0.0014	0.0143	7
WM45	mediumpurple3	-0.14	-0.24	0.33	0.4150	0.1585	0.0499	25
WM46	midnightblue	-0.10	0.30	-0.17	0.5670	0.0796	0.3190	76
WM47	navajowhite2	-0.11	0.29	-0.15	0.5226	0.0882	0.3690	17
WM48	orange	-0.10	0.30	-0.17	0.5662	0.0790	0.3180	48
WM49	orangered3	0.02	0.31	-0.28	0.9074	0.0691	0.0947	8
WM50	orangered4	0.14	-0.13	-0.01	0.4096	0.4414	0.9627	26
WM51	paleturquoise	-0.18	0.05	0.12	0.2808	0.7751	0.4959	37
WM52	palevioletred3	0.05	0.11	-0.14	0.7733	0.5299	0.4263	18
WM53	pink	0.28	-0.11	-0.15	0.0948	0.5387	0.3726	97
WM54	plum	0.10	0.14	-0.20	0.5763	0.4148	0.2308	8
WM55	plum1	0.29	-0.10	-0.17	0.0834	0.5747	0.3229	28
WM56	plum2	-0.12	-0.12	0.22	0.4678	0.4686	0.2053	21
WM57	plum3	0.00	0.00	0.00	0.9879	0.9991	0.9887	5
WM58	purple	-0.12	-0.07	0.16	0.4978	0.6998	0.3552	95
WM59	red	-0.14	0.17	-0.02	0.4232	0.3338	0.8879	105
WM60	royalblue	0.29	-0.09	-0.17	0.0846	0.5882	0.3171	60
WM61	saddlebrown	0.05	0.24	-0.25	0.7782	0.1570	0.1403	40
WM62	salmon	-0.10	-0.10	0.17	0.5698	0.5754	0.3261	86
WM63	salmon2	0.10	0.27	-0.32	0.5782	0.1067	0.0573	5
WM64	salmon4	-0.32	-0.20	0.45	0.0574	0.2436	0.0059	19
WM65	sienna3	-0.01	0.02	-0.01	0.9455	0.9046	0.9645	31
WM66	skyblue	-0.13	0.01	0.10	0.4674	0.9516	0.5648	41
WM67	skyblue1	-0.15	0.20	-0.04	0.3925	0.2489	0.8008	8
WM68	skyblue2	-0.25	-0.06	0.27	0.1479	0.7206	0.1160	11
WM69	skyblue3	-0.15	-0.15	0.25	0.3896	0.3940	0.1336	29
WM70	steelblue	0.10	0.13	-0.20	0.5534	0.4655	0.2490	38
WM71	tan	0.28	-0.10	-0.16	0.0924	0.5466	0.3618	86
WM72	thistle	-0.19	-0.17	0.31	0.2571	0.3343	0.0645	5
WM73	thistle1	-0.10	0.30	-0.17	0.5672	0.0798	0.3193	19
WM74	thistle2	-0.07	-0.16	0.20	0.7047	0.3414	0.2470	21
WM75	thistle3	-0.09	-0.35	0.38	0.6167	0.0355	0.0226	5
WM76	turquoise	-0.12	-0.12	0.20	0.5022	0.4919	0.2361	203
WM77	violet	-0.04	0.27	-0.20	0.8242	0.1073	0.2347	34
WM78	white	0.28	-0.11	-0.15	0.1040	0.5349	0.3957	46
WM79	yellow	-0.10	-0.10	0.17	0.5709	0.5721	0.3247	124
WM80	yellow4	0.05	-0.03	-0.02	0.7643	0.8636	0.9118	9
WM81	yellowgreen	-0.10	-0.09	0.17	0.5642	0.5920	0.3330	30

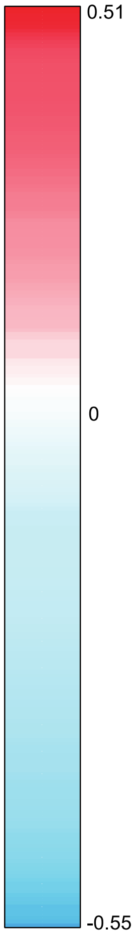

Supplementary Figure S3.

Pathway enrichment analysis and protein interaction networks for protein groups by analysis of variance (ANOVA).

A. Pathway enrichment analysis using the GO Biological Process database for protein groups identified by ANOVA. B. Pathway enrichment analysis using the Reactome pathway database for protein groups identified by ANOVA. The vertical axis shows the pathway names, and the bars on the horizontal axis represent the −log10 (*p*-value) of the corresponding pathways. Orange, sky blue, magenta, cyan, red and blue bars indicate the results for groups 1, 2, 3, 4, 5 and 6, respectively. Dashed lines in orange, magenta and red indicate *p*-values <0.05, <0.01 and <0.001, respectively. C. Protein network for proteins identified by ANOVA.

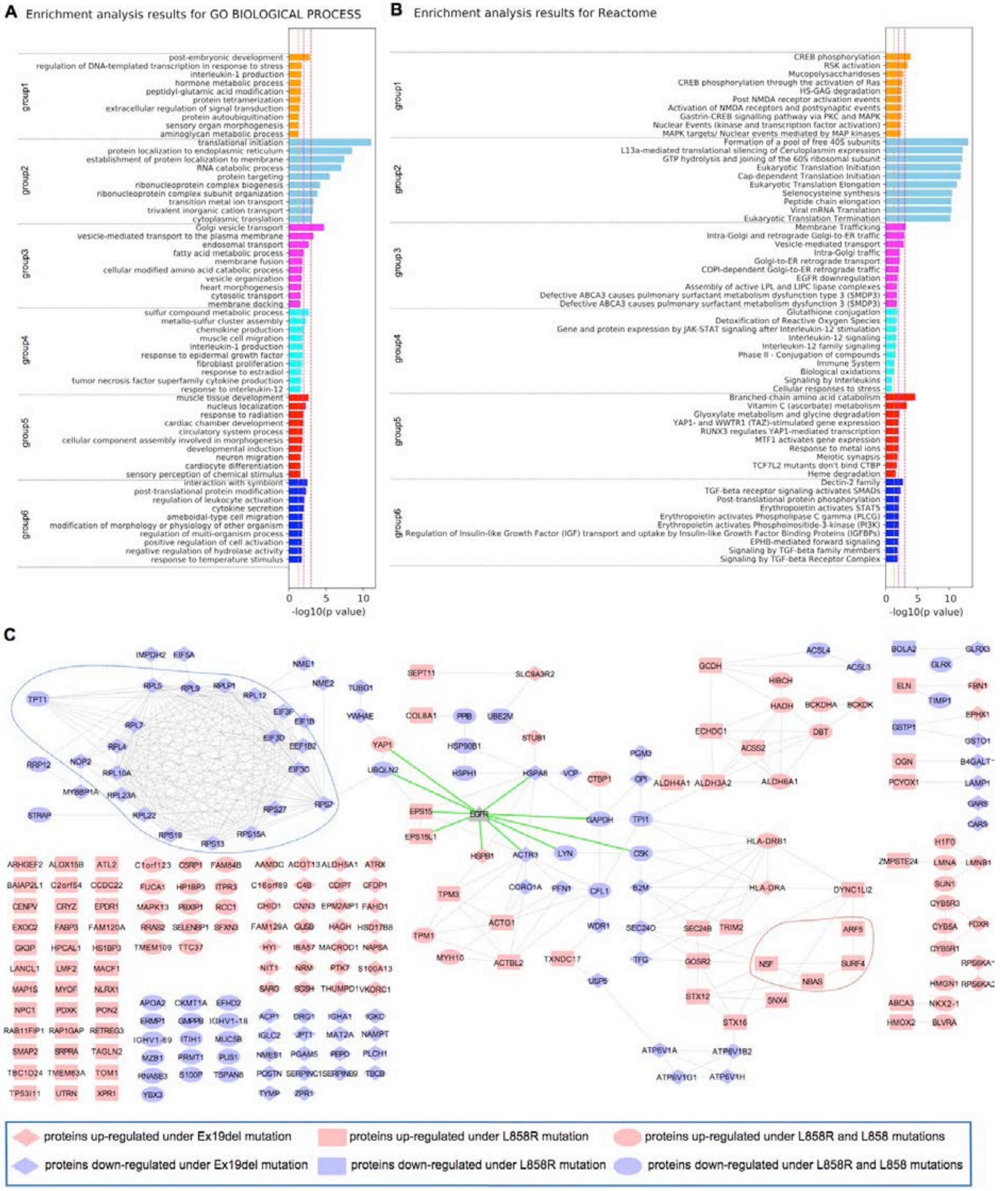

Supplementary Material. Properties of the focused 13 WGCNA module in Figure 3-5.

In each page, A. Boxplots of eigen protein profiles in the focused modules. The boxplot show the eigen protein expression abundance (the protein name is indicated in the plot title), and outlier data points indicate the outlier samples within each group. B. Histograms of expression abundance of the eigen protein for each sample group. Horizontal axis indicates abundance of the eigen protein and vertical axis indicates the absolute number of samples in each bin. C. Heatmap for the values of log2(abundance+1) for all membership proteins in the module normalize across the samples. We used R heatmap3 function with default settings to visualize the data.

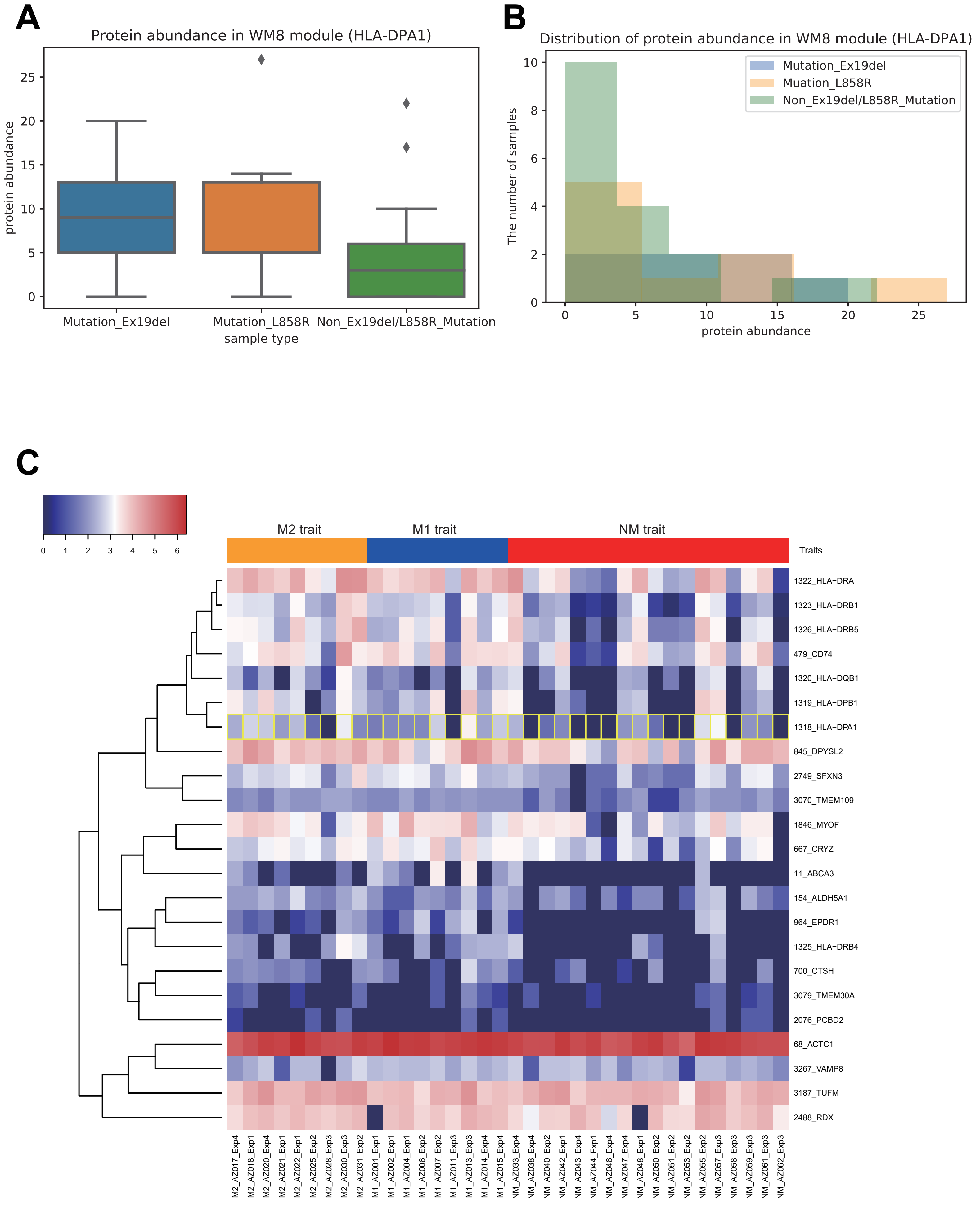
A
Protein abundance in WM8 module (HLA-DPA1)
protein abundance
sample type
Mutation_Ex19del
Mutation_L858R
Non_Ex19del/L858R_Mutation
B
Distribution of protein abundance in WM8 module (HLA-DPA1)
The number of samples
protein abundance
Mutation_Ex19del
Muation_L858R
Non_Ex19del/L858R_Mutation
C
M2 trait
M1 trait
NM trait
Traits
1322_HLA-DRA
1323_HLA-DRB1
1326_HLA-DRB5
479_CD74
1320_HLA-DQB1
1319_HLA-DPB1
1318_HLA-DPA1
845_DPYSL2
2749_SFXN3
3070_TMEM109
1846_MYOF
667_CRYZ
11_ABCA3
154_ALDH5A1
964_EPDR1
1325_HLA-DRB4
700_CTSH
3079_TMEM30A
2076_PCBD2
68_ACTC1
3267_VAMP8
3187_TUFM
2488_RDX

WM8

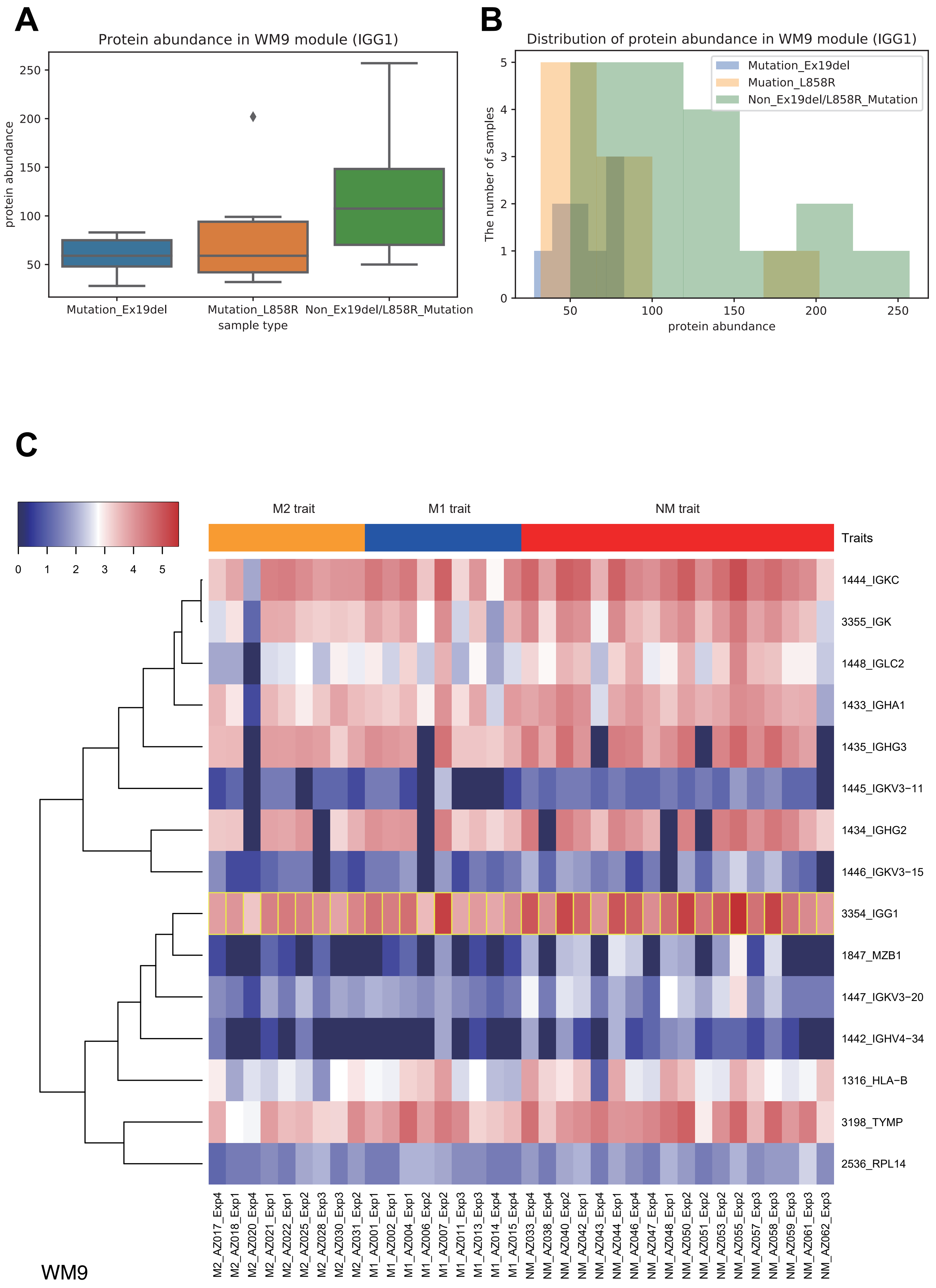
A
Protein abundance in WM9 module (IGG1)
protein abundance
250
200
150
100
50
Mutation_Ex19del
Mutation_L858R
Non_Ex19del/L858R_Mutation
sample type
B
Distribution of protein abundance in WM9 module (IGG1)
The number of samples
Mutation_Ex19del
Muation_L858R
Non_Ex19del/L858R_Mutation
protein abundance
C
M2 trait
M1 trait
NM trait
Traits
0 1 2 3 4 5
1444_IGKC
3355_IGK
1448_IGLC2
1433_IGHA1
1435_IGHG3
1445_IGKV3−11
1434_IGHG2
1446_IGKV3−15
3354_IGG1
1847_MZB1
1447_IGKV3−20
1442_IGHV4−34
1316_HLA-B
3198_TYMP
2536_RPL14
M2_AZ017_Exp4
M2_AZ018_Exp1
M2_AZ020_Exp4
M2_AZ021_Exp1
M2_AZ022_Exp1
M2_AZ025_Exp2
M2_AZ028_Exp3
M2_AZ030_Exp3
M2_AZ031_Exp2
M1_AZ001_Exp1
M1_AZ002_Exp1
M1_AZ004_Exp1
M1_AZ006_Exp2
M1_AZ007_Exp2
M1_AZ011_Exp3
M1_AZ013_Exp3
M1_AZ014_Exp4
M1_AZ015_Exp4
NM_AZ033_Exp4
NM_AZ038_Exp4
NM_AZ040_Exp2
NM_AZ042_Exp1
NM_AZ043_Exp4
NM_AZ044_Exp1
NM_AZ046_Exp4
NM_AZ047_Exp4
NM_AZ048_Exp1
NM_AZ050_Exp2
NM_AZ051_Exp2
NM_AZ053_Exp2
NM_AZ055_Exp2
NM_AZ057_Exp3
NM_AZ058_Exp3
NM_AZ059_Exp3
NM_AZ061_Exp3
NM_AZ062_Exp3
WM9

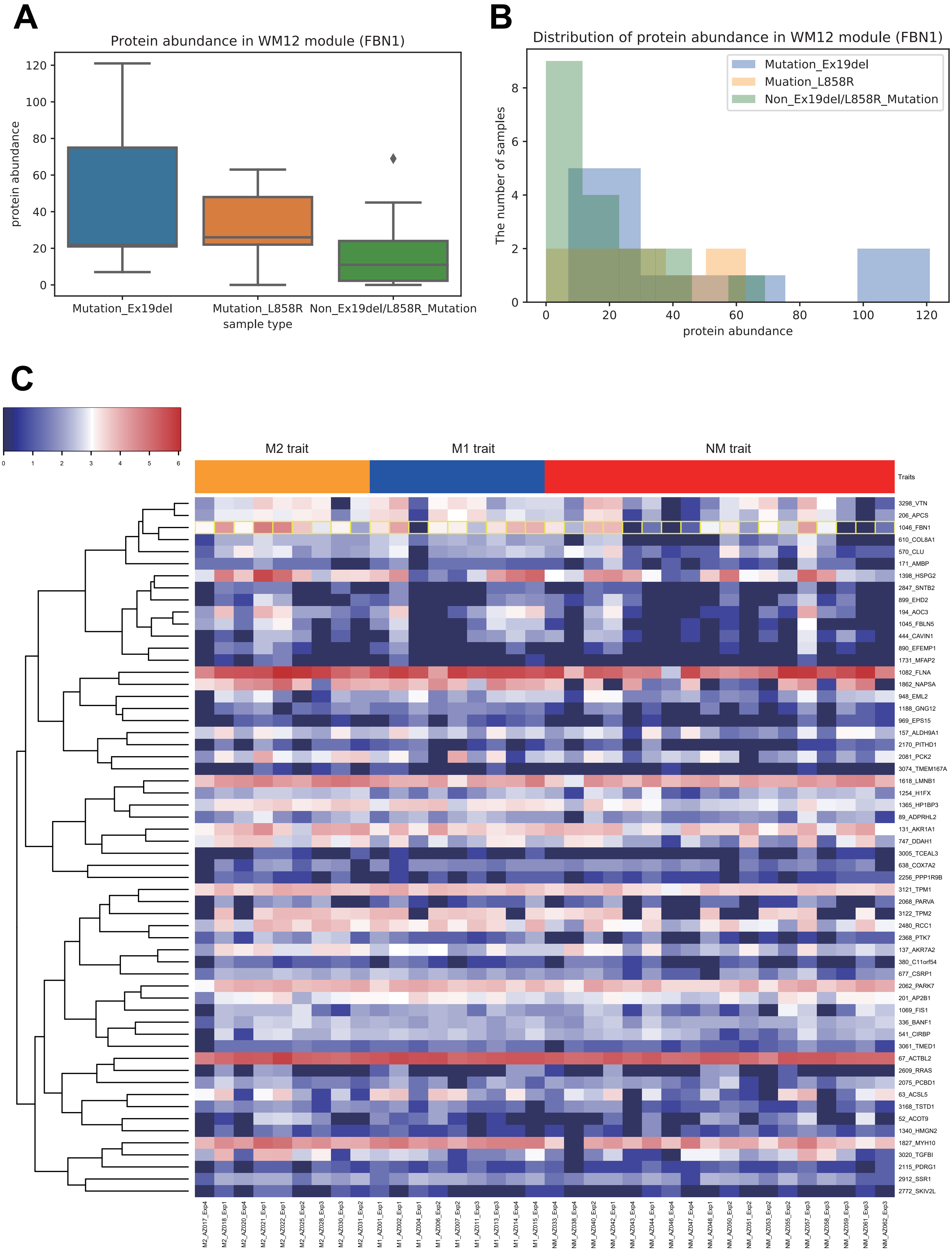
A
Protein abundance in WM12 module (FBN1)
protein abundance
120
100
80
60
40
20
0
Mutation_Ex19del
Mutation_L858R
Non_Ex19del/L858R_Mutation
sample type
B
Distribution of protein abundance in WM12 module (FBN1)
Mutation_Ex19del
Muation_L858R
Non_Ex19del/L858R_Mutation
The number of samples
8
6
4
2
0
0
20
40
60
80
100
120
protein abundance
C
0
1
2
3
4
5
6
M2 trait
M1 trait
NM trait
Traits
3298_VTN
206_APCS
1046_FBN1
610_COL8A1
570_CLU
171_AMBP
1398_HSPG2
2847_SNTB2
899_EHD2
194_AOC3
1045_FBLN5
444_CAVIN1
890_EFEMP1
1731_MFAP2
1082_FLNA
1862_NAPSA
948_EML2
1188_GNG12
969_EPS15
157_ALDH9A1
2170_PITHD1
2081_PCK2
3074_TMEM167A
1618_LMNB1
1254_H1FX
1365_HP1BP3
89_ADPRHL2
131_AKR1A1
747_DDAH1
3005_TCEAL3
638_COX7A2
2256_PPP1R9B
3121_TPM1
2068_PARVA
3122_TPM2
2480_RCC1
2368_PTK7
137_AKR7A2
380_C11orf54
677_CSRP1
2062_PARK7
201_AP2B1
1069_FIS1
336_BANF1
541_CIRBP
3061_TMED1
67_ACTBL2
2609_RRAS
2075_PCBD1
63_ACSL5
3168_TSTD1
52_ACOT9
1340_HMGN2
1827_MYH10
3020_TGFBI
2115_PDRG1
2912_SSR1
2772_SKIV2L

WM12

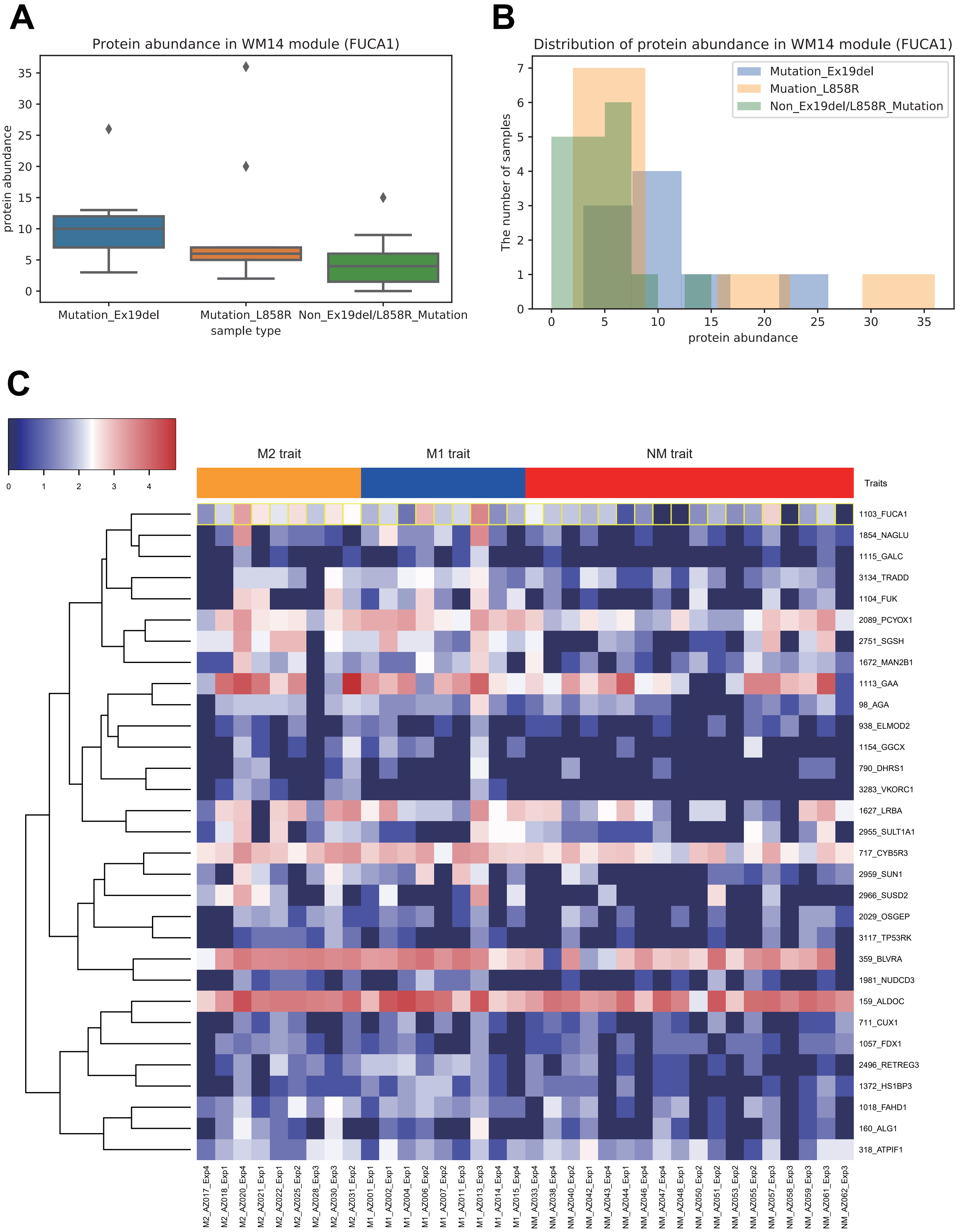

A
Protein abundance in WM14 module (FUCA1)
protein abundance
sample type
Mutation_Ex19del
Mutation_L858R
Non_Ex19del/L858R_Mutation
B
Distribution of protein abundance in WM14 module (FUCA1)
The number of samples
protein abundance
Mutation_Ex19del
Muation_L858R
Non_Ex19del/L858R_Mutation
C
M2 trait
M1 trait
NM trait
Traits
1103_FUCA1
1854_NAGLU
1115_GALC
3134_TRADD
1104_FUK
2089_PCYOX1
2751_SGSH
1672_MAN2B1
1113_GAA
98_AGA
938_ELMOD2
1154_GGCX
790_DHRS1
3283_VKORC1
1627_LRBA
2955_SULT1A1
717_CYB5R3
2959_SUN1
2966_SUSD2
2029_OSGEP
3117_TP53RK
359_BLVRA
1981_NUDCD3
159_ALDOC
711_CUX1
1057_FDX1
2496_RETREG3
1372_HS1BP3
1018_FAHD1
160_ALG1
318_ATPIF1

WM14

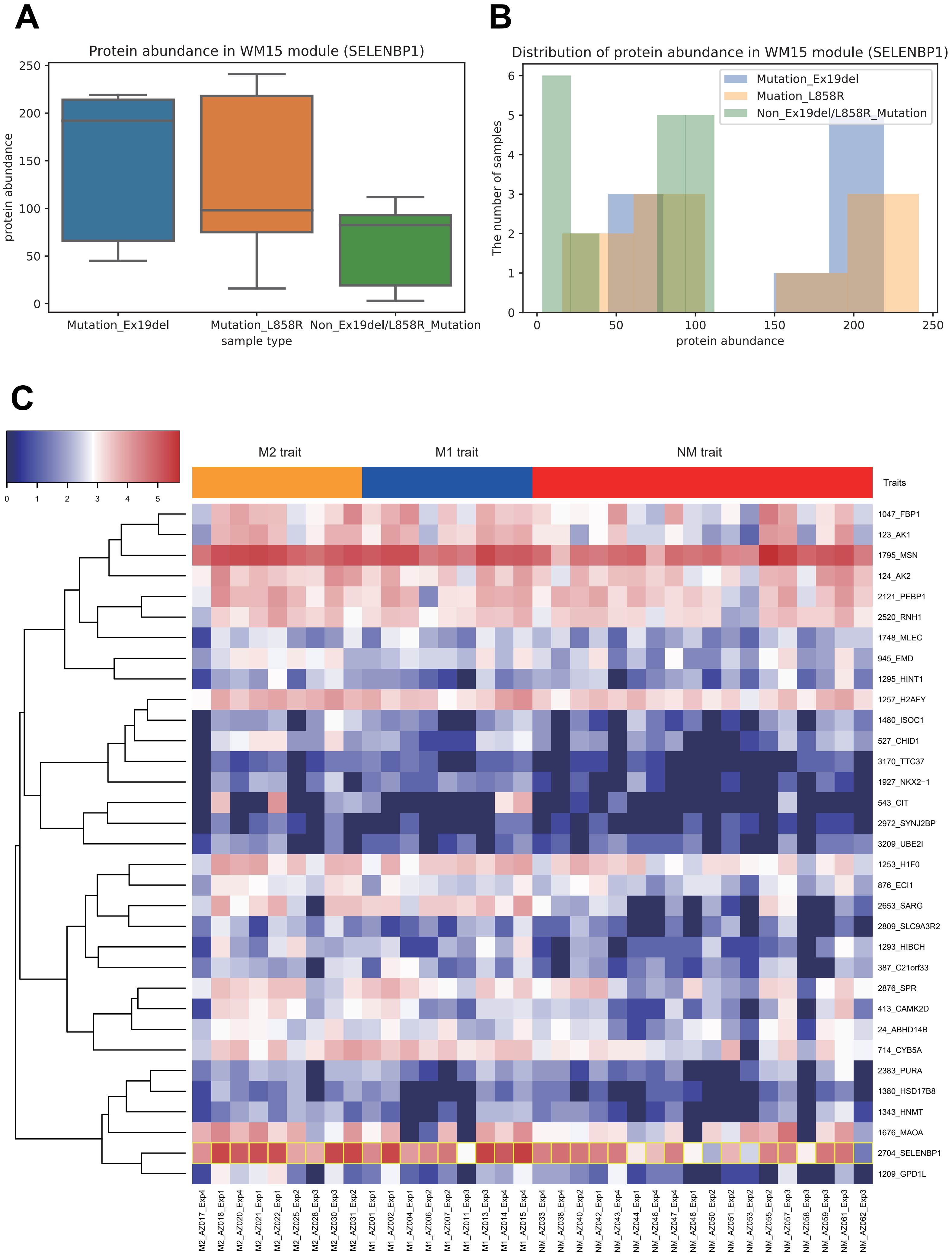
A
Protein abundance in WM15 module (SELENBP1)
protein abundance
Mutation_Ex19del
Mutation_L858R
Non_Ex19del/L858R_Mutation
sample type
B
Distribution of protein abundance in WM15 module (SELENBP1)
The number of samples
protein abundance
Mutation_Ex19del
Muation_L858R
Non_Ex19del/L858R_Mutation
C
M2 trait
M1 trait
NM trait
Traits
1047_FBP1
123_AK1
1795_MSN
124_AK2
2121_PEBP1
2520_RNH1
1748_MLEC
945_EMD
1295_HINT1
1257_H2AFY
1480_ISOC1
527_CHID1
3170_TTC37
1927_NKX2−1
543_CIT
2972_SYNJ2BP
3209_UBE2I
1253_H1F0
876_ECI1
2653_SARG
2809_SLC9A3R2
1293_HIBCH
387_C21orf33
2876_SPR
413_CAMK2D
24_ABHD14B
714_CYB5A
2383_PURA
1380_HSD17B8
1343_HNMT
1676_MAOA
2704_SELENBP1
1209_GPD1L
M2_AZ017_Exp4
M2_AZ018_Exp1
M2_AZ020_Exp4
M2_AZ021_Exp1
M2_AZ022_Exp1
M2_AZ025_Exp2
M2_AZ028_Exp3
M2_AZ030_Exp3
M2_AZ031_Exp2
M1_AZ001_Exp1
M1_AZ002_Exp1
M1_AZ004_Exp1
M1_AZ006_Exp2
M1_AZ007_Exp2
M1_AZ011_Exp3
M1_AZ013_Exp3
M1_AZ014_Exp4
M1_AZ015_Exp4
NM_AZ033_Exp4
NM_AZ038_Exp4
NM_AZ040_Exp2
NM_AZ042_Exp1
NM_AZ043_Exp4
NM_AZ044_Exp1
NM_AZ046_Exp4
NM_AZ047_Exp4
NM_AZ048_Exp1
NM_AZ050_Exp2
NM_AZ051_Exp2
NM_AZ053_Exp2
NM_AZ055_Exp2
NM_AZ057_Exp3
NM_AZ058_Exp3
NM_AZ059_Exp3
NM_AZ061_Exp3
NM_AZ062_Exp3

WM15

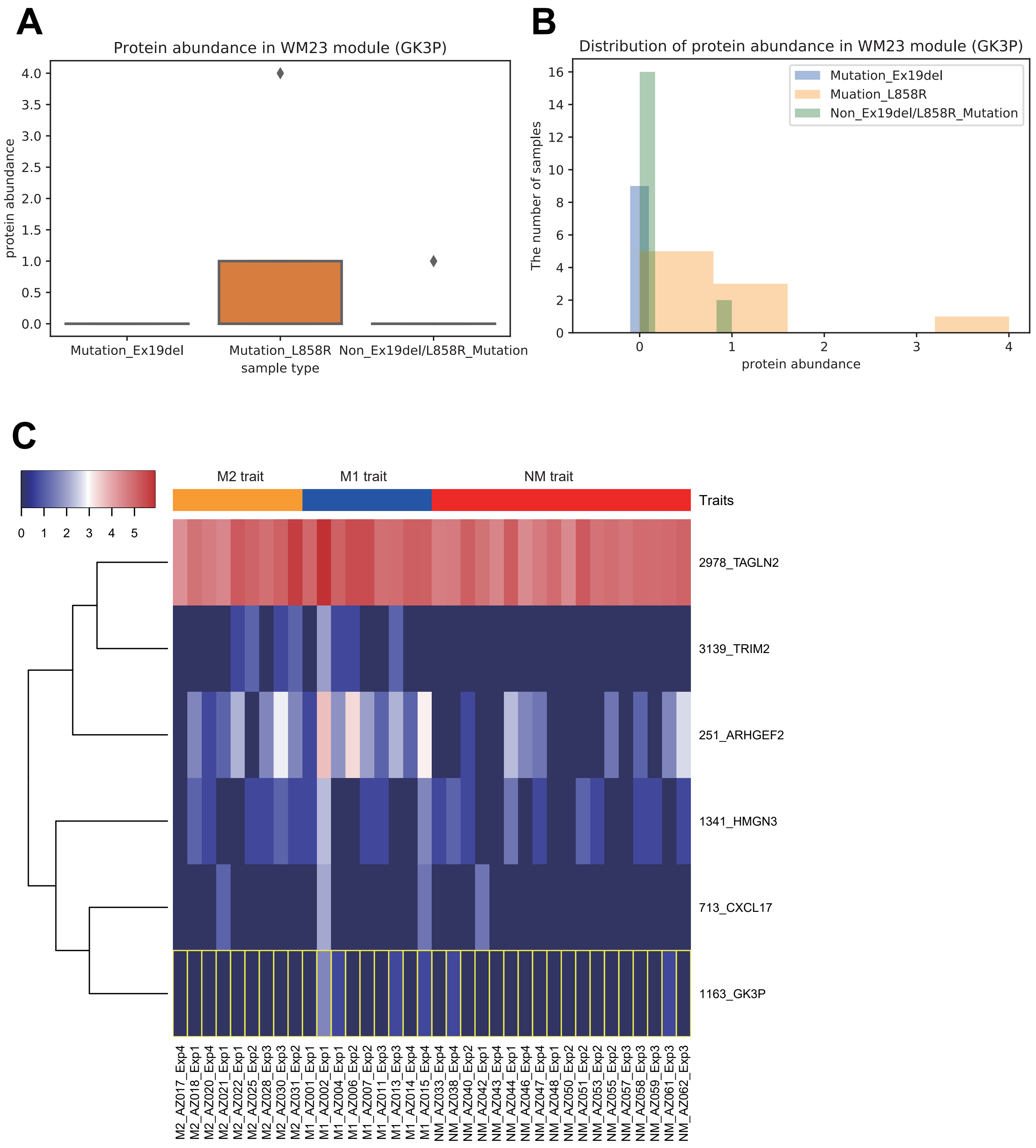
A
Protein abundance in WM23 module (GK3P)
protein abundance
sample type
Mutation_Ex19del
Mutation_L858R
Non_Ex19del/L858R_Mutation
B
Distribution of protein abundance in WM23 module (GK3P)
The number of samples
protein abundance
Mutation_Ex19del
Muation_L858R
Non_Ex19del/L858R_Mutation
C
M2 trait
M1 trait
NM trait
Traits
2978_TAGLN2
3139_TRIM2
251_ARHGEF2
1341_HMGN3
713_CXCL17
1163_GK3P

WM23

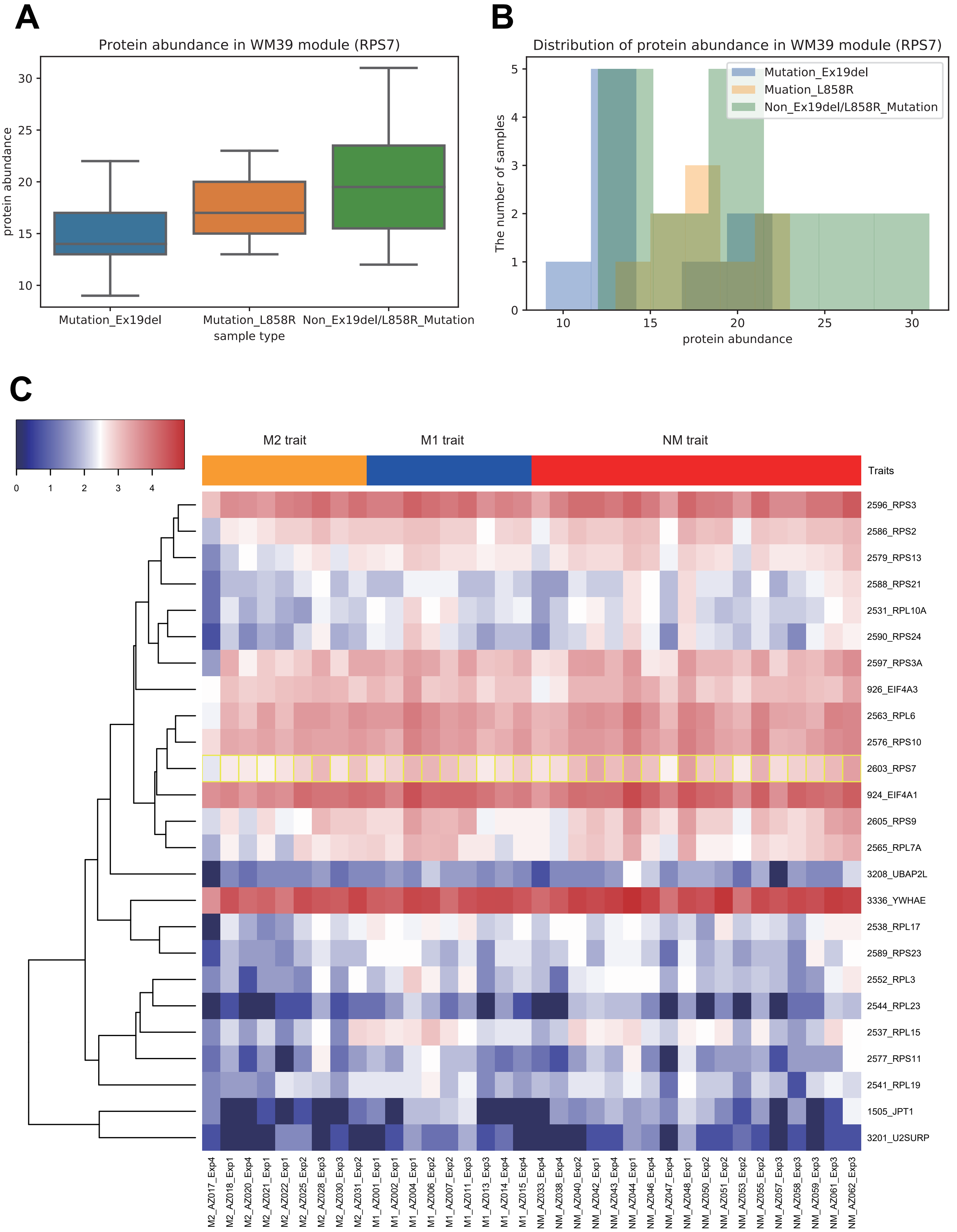
A
Protein abundance in WM39 module (RPS7)
protein abundance
sample type
Mutation_Ex19del
Mutation_L858R
Non_Ex19del/L858R_Mutation
B
Distribution of protein abundance in WM39 module (RPS7)
The number of samples
protein abundance
Mutation_Ex19del
Muation_L858R
Non_Ex19del/L858R_Mutation
C
M2 trait
M1 trait
NM trait
Traits
2596_RPS3
2586_RPS2
2579_RPS13
2588_RPS21
2531_RPL10A
2590_RPS24
2597_RPS3A
926_EIF4A3
2563_RPL6
2576_RPS10
2603_RPS7
924_EIF4A1
2605_RPS9
2565_RPL7A
3208_UBAP2L
3336_YWHAE
2538_RPL17
2589_RPS23
2552_RPL3
2544_RPL23
2537_RPL15
2577_RPS11
2541_RPL19
1505_JPT1
3201_U2SURP
M2_AZ017_Exp4
M2_AZ018_Exp1
M2_AZ020_Exp4
M2_AZ021_Exp1
M2_AZ022_Exp1
M2_AZ025_Exp2
M2_AZ028_Exp3
M2_AZ030_Exp3
M2_AZ031_Exp2
M1_AZ001_Exp1
M1_AZ002_Exp1
M1_AZ004_Exp1
M1_AZ006_Exp2
M1_AZ007_Exp2
M1_AZ011_Exp3
M1_AZ013_Exp3
M1_AZ014_Exp4
M1_AZ015_Exp4
NM_AZ033_Exp4
NM_AZ038_Exp4
NM_AZ040_Exp2
NM_AZ042_Exp1
NM_AZ043_Exp4
NM_AZ044_Exp1
NM_AZ046_Exp4
NM_AZ047_Exp4
NM_AZ048_Exp1
NM_AZ050_Exp2
NM_AZ051_Exp2
NM_AZ053_Exp2
NM_AZ055_Exp2
NM_AZ057_Exp3
NM_AZ058_Exp3
NM_AZ059_Exp3
NM_AZ061_Exp3
NM_AZ062_Exp3

WM39

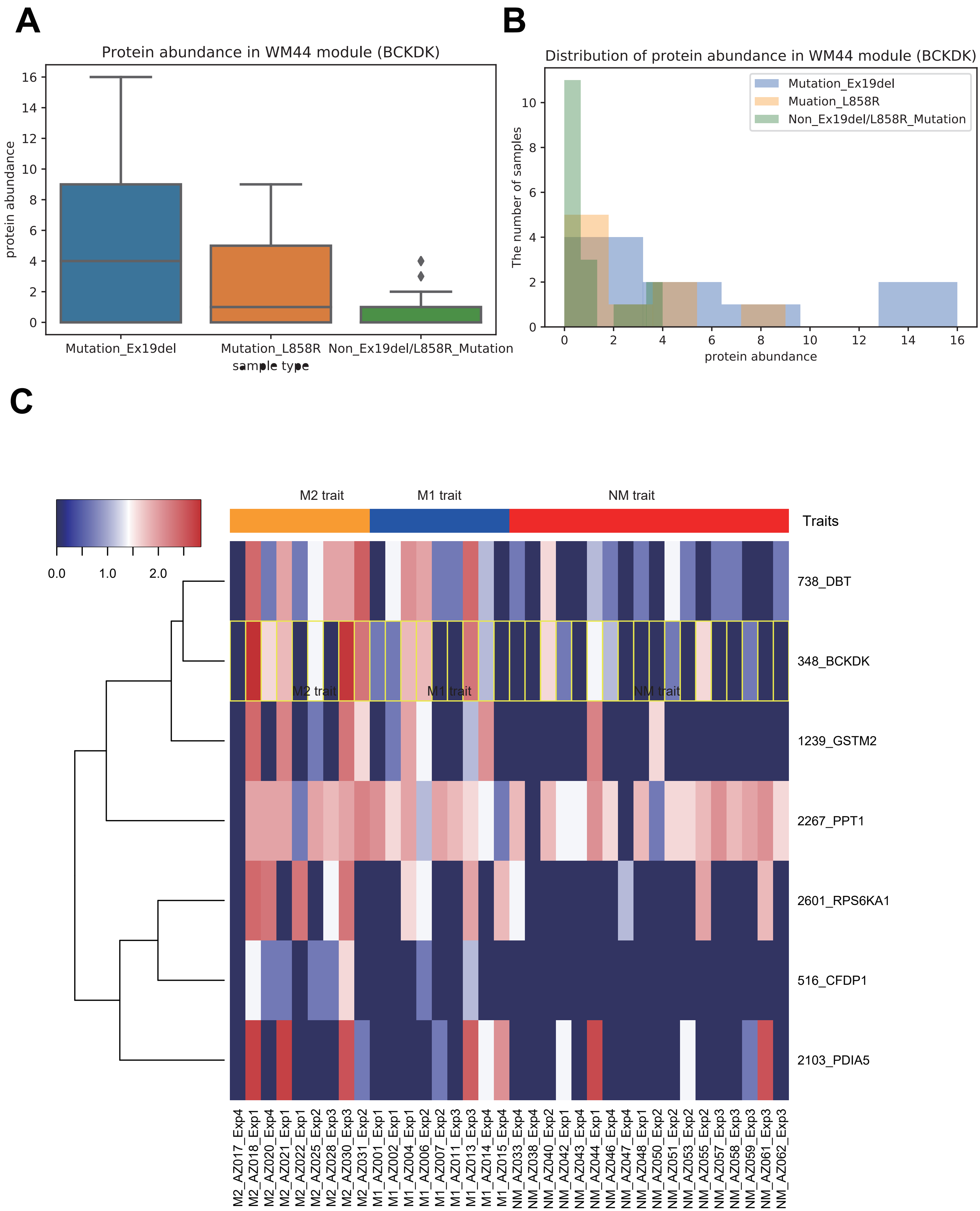
A
Protein abundance in WM44 module (BCKDK)
protein abundance
Mutation_Ex19del
Mutation_L858R
Non_Ex19del/L858R_Mutation
sample type
B
Distribution of protein abundance in WM44 module (BCKDK)
Mutation_Ex19del
Muation_L858R
Non_Ex19del/L858R_Mutation
The number of samples
protein abundance
C
M2 trait
M1 trait
NM trait
Traits
0.0
1.0
2.0
738_DBT
348_BCKDK
1239_GSTM2
2267_PPT1
2601_RPS6KA1
516_CFDP1
2103_PDIA5
M2_AZ017_Exp4
M2_AZ018_Exp1
M2_AZ020_Exp4
M2_AZ021_Exp1
M2_AZ022_Exp1
M2_AZ025_Exp2
M2_AZ028_Exp3
M2_AZ030_Exp3
M2_AZ031_Exp2
M1_AZ001_Exp1
M1_AZ002_Exp1
M1_AZ004_Exp1
M1_AZ006_Exp2
M1_AZ007_Exp2
M1_AZ011_Exp3
M1_AZ013_Exp3
M1_AZ014_Exp4
M1_AZ015_Exp4
NM_AZ033_Exp4
NM_AZ038_Exp4
NM_AZ040_Exp2
NM_AZ042_Exp1
NM_AZ043_Exp4
NM_AZ044_Exp1
NM_AZ046_Exp4
NM_AZ047_Exp4
NM_AZ048_Exp1
NM_AZ050_Exp2
NM_AZ051_Exp2
NM_AZ053_Exp2
NM_AZ055_Exp2
NM_AZ057_Exp3
NM_AZ058_Exp3
NM_AZ059_Exp3
NM_AZ061_Exp3
NM_AZ062_Exp3

WM44

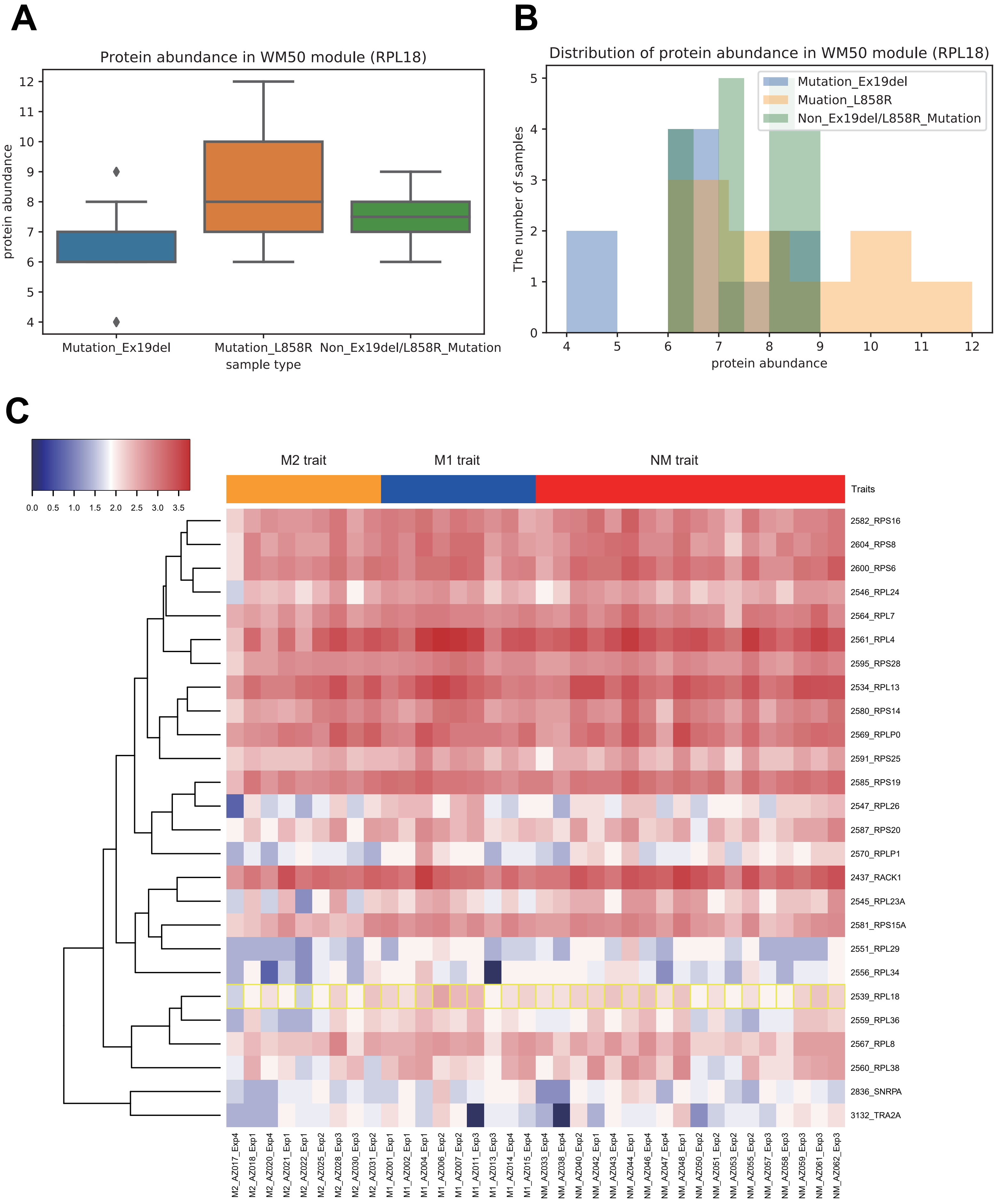
A
Protein abundance in WM50 module (RPL18)
protein abundance
sample type
Mutation_Ex19del
Mutation_L858R
Non_Ex19del/L858R_Mutation
B
Distribution of protein abundance in WM50 module (RPL18)
Mutation_Ex19del
Muation_L858R
Non_Ex19del/L858R_Mutation
The number of samples
protein abundance
C
M2 trait
M1 trait
NM trait
Traits
2582_RPS16
2604_RPS8
2600_RPS6
2546_RPL24
2564_RPL7
2561_RPL4
2595_RPS28
2534_RPL13
2580_RPS14
2569_RPLP0
2591_RPS25
2585_RPS19
2547_RPL26
2587_RPS20
2570_RPLP1
2437_RACK1
2545_RPL23A
2581_RPS15A
2551_RPL29
2556_RPL34
2539_RPL18
2559_RPL36
2567_RPL8
2560_RPL38
2836_SNRPA
3132_TRA2A
M2_AZ017_Exp4
M2_AZ018_Exp1
M2_AZ020_Exp4
M2_AZ021_Exp1
M2_AZ022_Exp1
M2_AZ025_Exp2
M2_AZ028_Exp3
M2_AZ030_Exp3
M2_AZ031_Exp2
M1_AZ001_Exp1
M1_AZ002_Exp1
M1_AZ004_Exp1
M1_AZ006_Exp2
M1_AZ007_Exp2
M1_AZ011_Exp3
M1_AZ013_Exp3
M1_AZ014_Exp4
M1_AZ015_Exp4
NM_AZ033_Exp4
NM_AZ038_Exp4
NM_AZ040_Exp2
NM_AZ042_Exp1
NM_AZ043_Exp4
NM_AZ044_Exp1
NM_AZ046_Exp4
NM_AZ047_Exp4
NM_AZ048_Exp1
NM_AZ050_Exp2
NM_AZ051_Exp2
NM_AZ053_Exp2
NM_AZ055_Exp2
NM_AZ057_Exp3
NM_AZ058_Exp3
NM_AZ059_Exp3
NM_AZ061_Exp3
NM_AZ062_Exp3

WM50

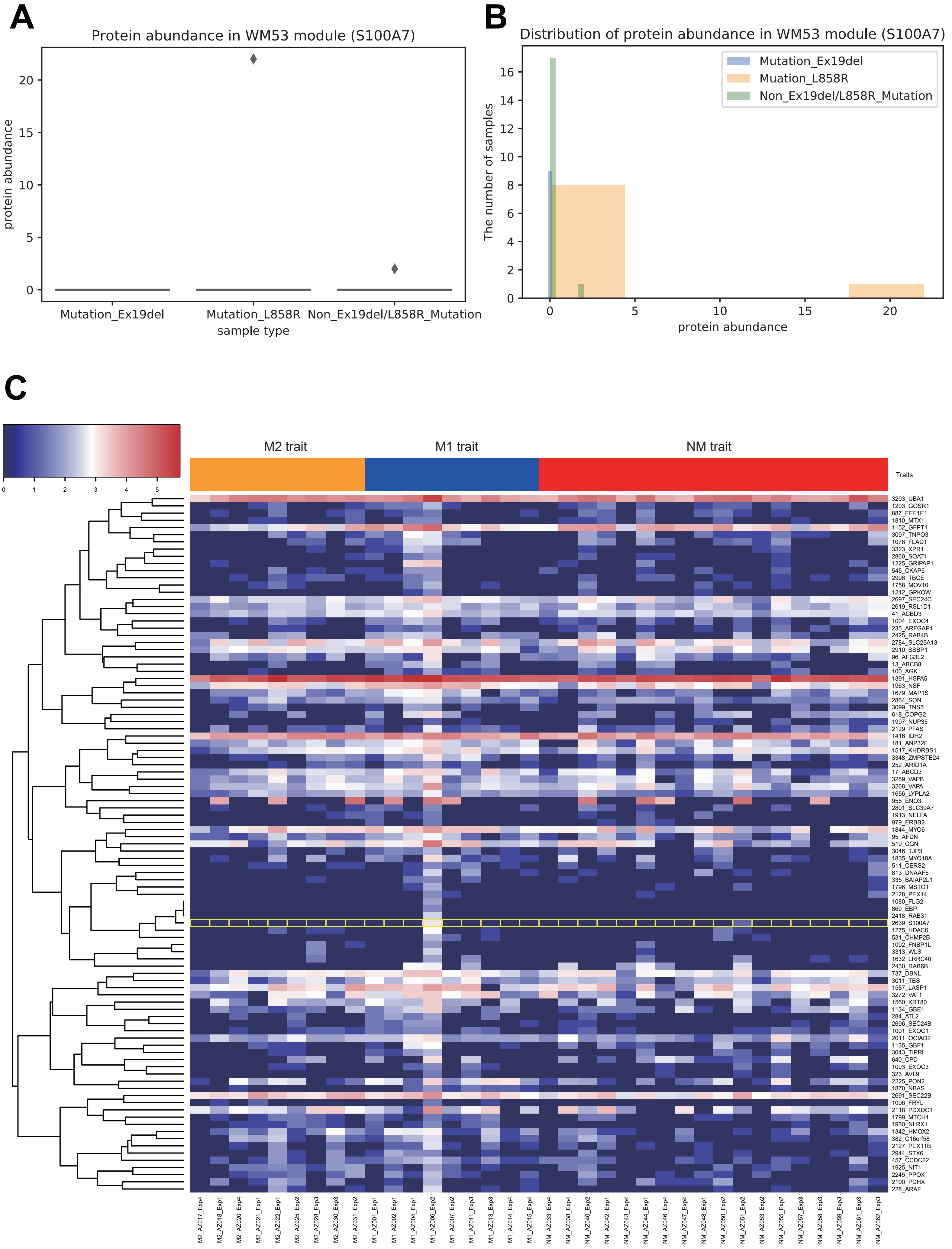
A
Protein abundance in WM53 module (S100A7)
protein abundance
0
5
10
15
20
Mutation_Ex19del
Mutation_L858R
Non_Ex19del/L858R_Mutation
sample type
B
Distribution of protein abundance in WM53 module (S100A7)
Mutation_Ex19del
Muation_L858R
Non_Ex19del/L858R_Mutation
The number of samples
0
2
4
6
8
10
12
14
16
protein abundance
C
0
1
2
3
4
5
M2 trait
M1 trait
NM trait
Traits
3203_UBA1
1203_GOSR1
887_EEF1E1
1810_MTX1
1152_GFPT1
3097_TNPO3
1078_FLAD1
3323_XPR1
2860_SOAT1
1225_GRIPAP1
545_CKAP5
2998_TBCE
1758_MOV10
1212_GPKOW
2697_SEC24C
2619_RSL1D1
41_ACBD3
1004_EXOC4
235_ARFGAP1
2425_RAB4B
2784_SLC25A13
2910_SSBP1
96_AFG3L2
13_ABCB8
100_AGK
1391_HSPA5
1963_NSF
1679_MAP1S
2864_SON
3099_TNS3
618_COPG2
1997_NUP35
2129_PFAS
1416_IDH2
181_ANP32E
1517_KHDRBS1
3348_ZMPSTE24
252_ARID1A
17_ABCD3
3269_VAPB
3268_VAPA
1656_LYPLA2
955_ENO3
2801_SLC39A7
1913_NELFA
979_ERBB2
1844_MYO6
95_AFDN
519_CGN
3046_TJP3
1835_MYO18A
511_CERS2
813_DNAAF5
335_BAIAP2L1
1796_MSTO1
2128_PEX14
1080_FLG2
869_EBP
2418_RAB31
2639_S100A7
1275_HDAC6
531_CHMP2B
1092_FNBP1L
3313_WLS
1632_LRRC40
2430_RAB6B
737_DBNL
3011_TES
1587_LASP1
3272_VAT1
1560_KRT80
1134_GBE1
284_ATL2
2696_SEC24B
1001_EXOC1
2011_OCIAD2
1135_GBF1
3043_TIPRL
640_CPD
1003_EXOC3
323_AVL9
2225_PON2
1870_NBAS
2691_SEC22B
1096_FRYL
2118_PDXDC1
1799_MTCH1
1930_NLRX1
1342_HMOX2
382_C16orf58
2127_PEX11B
2944_STX6
457_CCDC22
1925_NIT1
2245_PPOX
2100_PDHX
228_ARAF
M2_AZ017_Exp4
M2_AZ018_Exp1
M2_AZ020_Exp4
M2_AZ021_Exp1
M2_AZ022_Exp1
M2_AZ025_Exp2
M2_AZ028_Exp3
M2_AZ030_Exp3
M2_AZ031_Exp2
M1_AZ001_Exp1
M1_AZ002_Exp1
M1_AZ004_Exp1
M1_AZ006_Exp2
M1_AZ007_Exp2
M1_AZ011_Exp3
M1_AZ013_Exp3
M1_AZ014_Exp4
M1_AZ015_Exp4
NM_AZ033_Exp4
NM_AZ038_Exp4
NM_AZ040_Exp2
NM_AZ042_Exp1
NM_AZ043_Exp4
NM_AZ044_Exp1
NM_AZ046_Exp4
NM_AZ047_Exp4
NM_AZ048_Exp1
NM_AZ050_Exp2
NM_AZ051_Exp2
NM_AZ053_Exp2
NM_AZ055_Exp2
NM_AZ057_Exp3
NM_AZ058_Exp3
NM_AZ059_Exp3
NM_AZ061_Exp3
NM_AZ062_Exp3

WM53

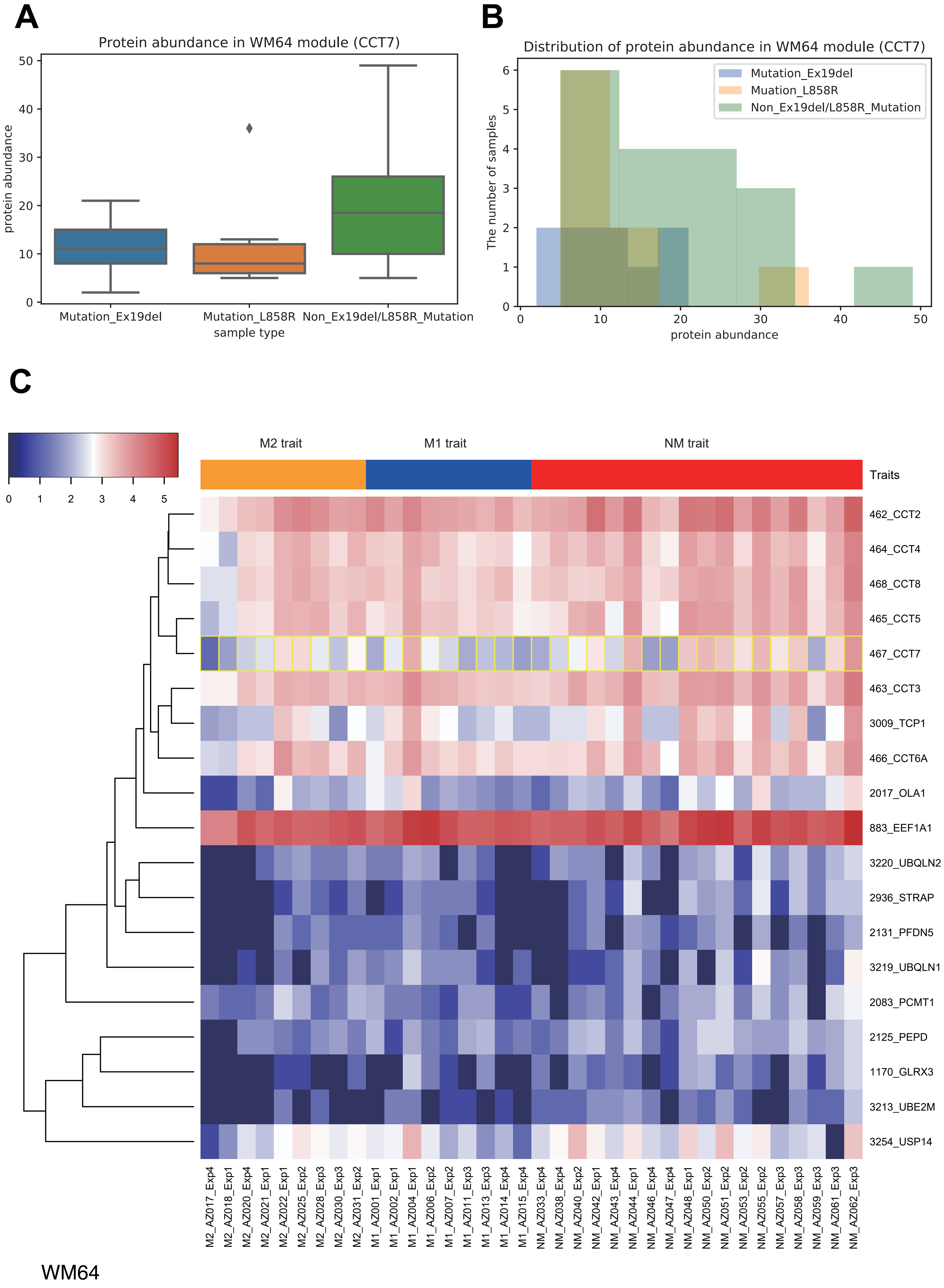
A
Protein abundance in WM64 module (CCT7)
protein abundance
Mutation_Ex19del
Mutation_L858R
Non_Ex19del/L858R_Mutation
sample type
B
Distribution of protein abundance in WM64 module (CCT7)
The number of samples
Mutation_Ex19del
Muation_L858R
Non_Ex19del/L858R_Mutation
protein abundance
C
M2 trait
M1 trait
NM trait
Traits
462_CCT2
464_CCT4
468_CCT8
465_CCT5
467_CCT7
463_CCT3
3009_TCP1
466_CCT6A
2017_OLA1
883_EEF1A1
3220_UBQLN2
2936_STRAP
2131_PFDN5
3219_UBQLN1
2083_PCMT1
2125_PEPD
1170_GLRX3
3213_UBE2M
3254_USP14
WM64

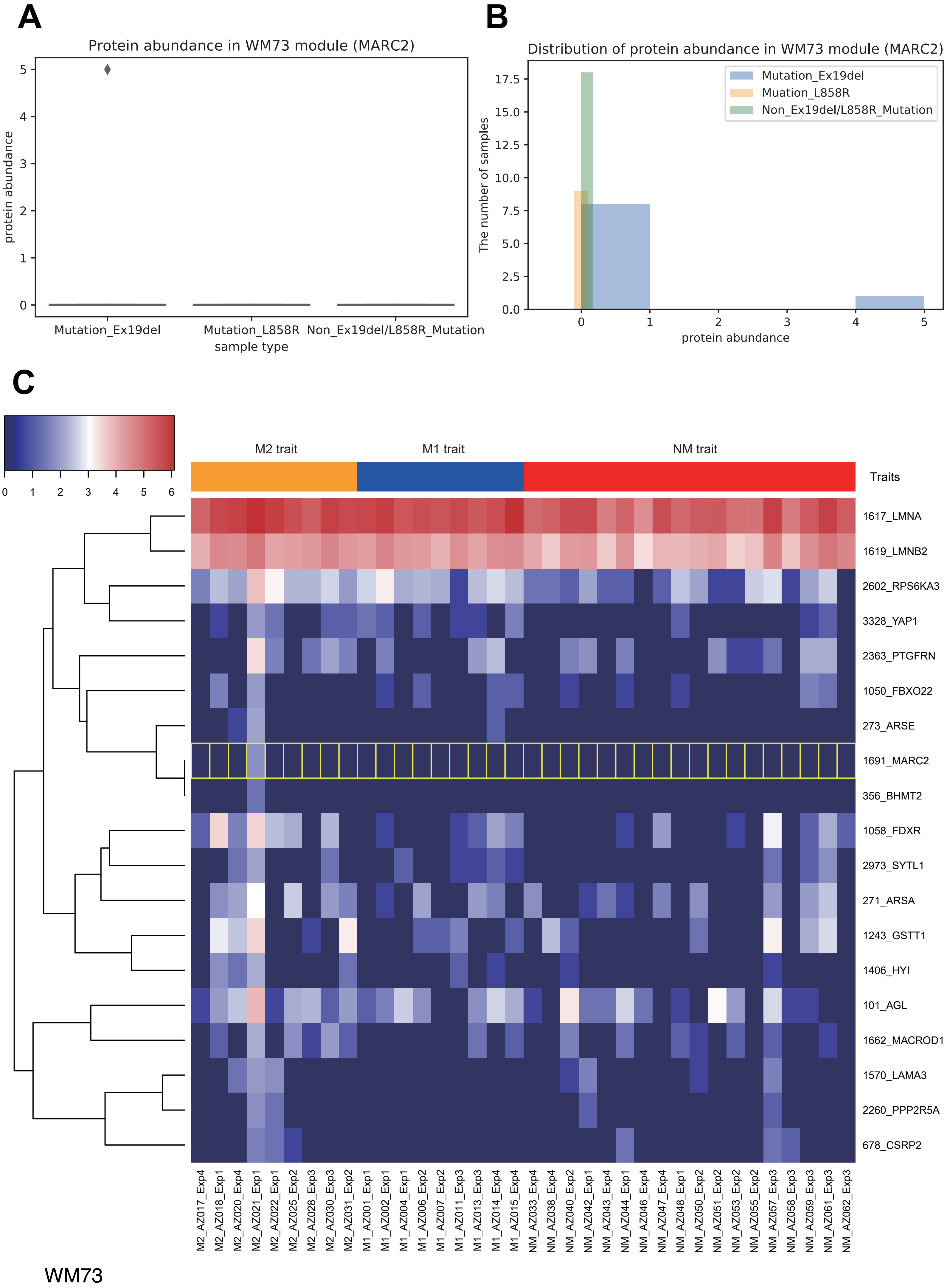
A
Protein abundance in WM73 module (MARC2)
protein abundance
sample type
Mutation_Ex19del
Mutation_L858R
Non_Ex19del/L858R_Mutation
B
Distribution of protein abundance in WM73 module (MARC2)
The number of samples
protein abundance
Mutation_Ex19del
Muation_L858R
Non_Ex19del/L858R_Mutation
C
M2 trait
M1 trait
NM trait
Traits
1617_LMNA
1619_LMNB2
2602_RPS6KA3
3328_YAP1
2363_PTGFRN
1050_FBXO22
273_ARSE
1691_MARC2
356_BHMT2
1058_FDXR
2973_SYTL1
271_ARSA
1243_GSTT1
1406_HYI
101_AGL
1662_MACROD1
1570_LAMA3
2260_PPP2R5A
678_CSRP2
M2_AZ017_Exp4
M2_AZ018_Exp1
M2_AZ020_Exp4
M2_AZ021_Exp1
M2_AZ022_Exp1
M2_AZ025_Exp2
M2_AZ028_Exp3
M2_AZ030_Exp3
M2_AZ031_Exp2
M1_AZ001_Exp1
M1_AZ002_Exp1
M1_AZ004_Exp1
M1_AZ006_Exp2
M1_AZ007_Exp2
M1_AZ011_Exp3
M1_AZ013_Exp3
M1_AZ014_Exp4
M1_AZ015_Exp4
NM_AZ033_Exp4
NM_AZ038_Exp4
NM_AZ040_Exp2
NM_AZ042_Exp1
NM_AZ043_Exp4
NM_AZ044_Exp1
NM_AZ046_Exp4
NM_AZ047_Exp4
NM_AZ048_Exp1
NM_AZ050_Exp2
NM_AZ051_Exp2
NM_AZ053_Exp2
NM_AZ055_Exp2
NM_AZ057_Exp3
NM_AZ058_Exp3
NM_AZ059_Exp3
NM_AZ061_Exp3
NM_AZ062_Exp3
WM73

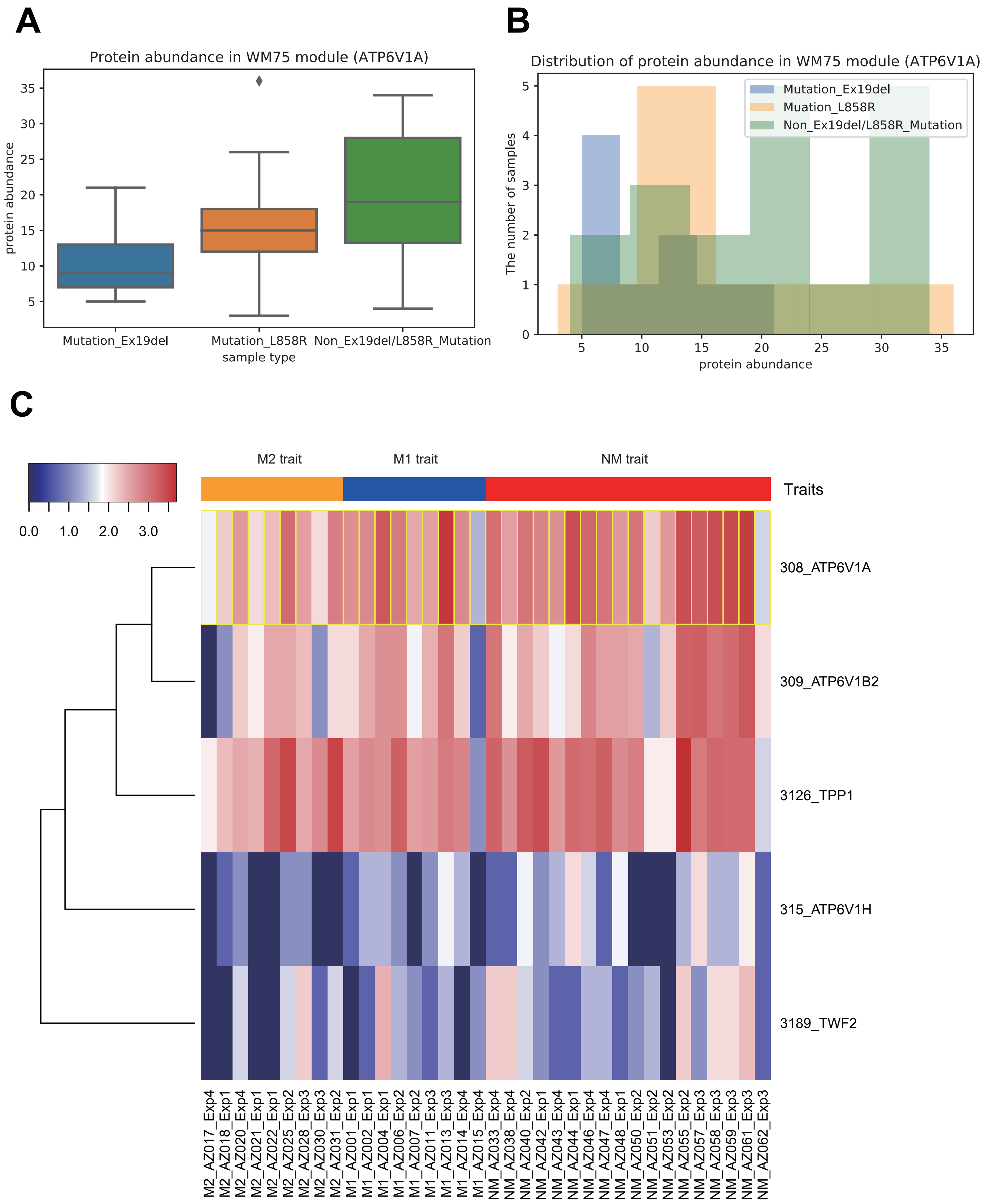
A
Protein abundance in WM75 module (ATP6V1A)
protein abundance
sample type
Mutation_Ex19del
Mutation_L858R
Non_Ex19del/L858R_Mutation
B
Distribution of protein abundance in WM75 module (ATP6V1A)
The number of samples
protein abundance
Mutation_Ex19del
Muation_L858R
Non_Ex19del/L858R_Mutation
C
M2 trait
M1 trait
NM trait
Traits
308_ATP6V1A
309_ATP6V1B2
3126_TPP1
315_ATP6V1H
3189_TWF2
M2_AZ017_Exp4
M2_AZ018_Exp1
M2_AZ020_Exp4
M2_AZ021_Exp1
M2_AZ022_Exp1
M2_AZ025_Exp2
M2_AZ028_Exp3
M2_AZ030_Exp3
M2_AZ031_Exp2
M1_AZ001_Exp1
M1_AZ002_Exp1
M1_AZ004_Exp1
M1_AZ006_Exp2
M1_AZ007_Exp2
M1_AZ011_Exp3
M1_AZ013_Exp3
M1_AZ014_Exp4
M1_AZ015_Exp4
NM_AZ033_Exp4
NM_AZ038_Exp4
NM_AZ040_Exp2
NM_AZ042_Exp1
NM_AZ043_Exp4
NM_AZ044_Exp1
NM_AZ046_Exp4
NM_AZ047_Exp4
NM_AZ048_Exp1
NM_AZ050_Exp2
NM_AZ051_Exp2
NM_AZ053_Exp2
NM_AZ055_Exp2
NM_AZ057_Exp3
NM_AZ058_Exp3
NM_AZ059_Exp3
NM_AZ061_Exp3
NM_AZ062_Exp3

WM75
